# Supplementary material for: MICU1 controls cristae junction and spatially anchors mitochondrial Ca2+ uniporter complex
Source: Nat Commun. 2019 Aug 19;10:3732. doi: 10.1038/s41467-019-11692-x (PMC6700202; doi:10.1038/s41467-019-11692-x)
Supplement: Supplementary file 1 — Supplementary Information [file 41467_2019_11692_MOESM1_ESM.pdf]

MICU1 controls cristae junction and spatially anchors mitochondrial Ca^{2+} uniporter complex

Benjamin Gottschalk[1], Christiane Klec[1], Gerd Leitinger[2], Eva Bernhart[1], Rene Rost[1], Helmut Bischof[1], Corina Madreiter-Sokolowski[1], Snjezana Radulovic[1,2], Emrah Eroglu[1], Wolfgang Sattler[1,3], Markus Waldeck-Weiermair[1], Roland Malli[1,3] & Wolfgang F. Graier[1,3,4]

Supplementary Table 1:

	Target	Sequence
si RNA	MICU1 si	5’-GCAGCUCAAGAAGCACUUCAAdTdT-3’
	nonCDSMICU1 si	5’-AGAAGUCUGUGAUGAUAAAdTdT-3’
	OPA1 si	5’-GUUAUCAGUCUGAGCCAGGdTdT-3’
	PRMT1 si	5’-CGUCAAACCCAACAAGUUAdTdT-3’
	MCU si 1	5’-GCCAGAGACAGACAAUACUdTdT-3’
	MCU si 2	5’-GGAAAGGGAGCUUAUUGAAdTdT-3’
	EMRE si	5’-GAACUUUGCUGCUCUACUUdTdT-3’
	taffazin si	5’-CCUACAGCUGCUUCUGGACCAAGUAdTdT-3’
	control si	5’-UUCUCCGAACGUGUCACGU-3’
cloning primer	MICU1 pos. 1 forward	5'-CCCGGATCCATGTTTCGTCTGAACTCACT-3'
	MICU1 pos. 1332 reverse	5'-GGTTGAATTCCATCAGCCGTTGCTTCATGAT-3'
	MICU1 pos. 210 reverse	5'-GGTTGAATTCTTTAGGTTGTCTACACATGG-3'
	MICU1 pos. 420 reverse	5'-GGTTGGATCCTTCAAGGTGGCAAAATATCGG-3'
	ROMO1 pos. 1 forward	5'-AAATCTAGACTCGAGCATGCCGGTGGCCGTGGGT-3'
	ROMO1 position 255 reverse	5'-CATACTCGAGTCTAGACGGATGCCCATCCCAATGG-3'
	mCherry for	5'-CGA-GAATTCATGGTGAGCAAGG-3'
	mCherry-myc P1 rev	5'-TGAGTTTTTGTTCGGATCCCTTGTACAGCTCGTCCATGCC-3'
	mCherry-myc P2 rev	5'-CGACGGCGCTATTCAGATCCTCTTCTGAGATGAGTTTTTGTTCGGATCCC-3'
	mCherry-myc-HIS P3 rev	5'-CCCTCTAGATTAATGGTGATGGTGATGATGGTCGACGGCGCTATTCAGATCC-3'
	mCherry HIS XbaI P4 rev	5'-CCCTCTAGATTAATGGTGATGG-3'
qRT-PCR	MICU1-for	5’-CAGGTTCAGAGCATCATTCG-3’
	MICU1-rev	5’-GAACACAAGCCAGACTTGAG-3’
	TAF-for	5’- GGAGAACAAGTCGGCTGTGGAG-3’
	TAF-rev	5’- GGAGCTGCTCTGCCTGAGTCTT-3’

The siRNAs and primers used for experiments: All siRNA used were purchased form Microsynth (Switzerland). Primer for cloning and qRT-PCR were purchased from Invitrogen (Vienna, Austria).

[1] Gottfried Schatz Research Center, Molecular Biology and Biochemistry, Medical University of Graz, Neue Stiftingtalstraße 6/6, 8010 Graz, Austria
[2] Gottfried Schatz Research Center, Cell Biology, Histology and Embryology, Medical University of Graz, Neue Stiftingtalstraße 6/2, 8010 Graz, Austria
[3] BioTechMed Graz, Austria
[4] Correspondence and requests for materials should be addressed to W.F.G. (wolfgang.graier@medunigraz.at).

Supplementary Table 2:

Figure	Reference label	Analysis label	Dilatation iterations	Erosion iterations	Fill holes
2b	EMRE-mCherry	MICU1-YFP	2	6	no
2g, 3a&b, supple. 12, supple. 13	MTR-FM	MICU1$^{1-70}$-GFP, MICU1$^{1-140}$-GFP, MICU1-YFP, ΔC-MICU1-YFP, MICU1-F-YFP, MICU1-K-YFP, MICU1-EF-YFP	2	6	no
4f	MTR-CMXROS	Cytochrome-c	3	3	no
6b&d, 7b&d, 8a, supple. 24, supple. 26, supple. 29, supple. 33	MICU1-YFP	MCU-mCherry, EMRE-mCherry, MTR-FM, UCP2-mCherry	1	6	yes
8c, supple. 25, supple. 34	MTG	UCP2-mCherry, MCU-mCherry	4	2	no

IBM association index detailed parameter. Summary of different directly comparable experiments and the analysis parameter (dilatation, erosion, fill holes) with references to figures, reference and analytic labels.

Supplementary Figure 1:

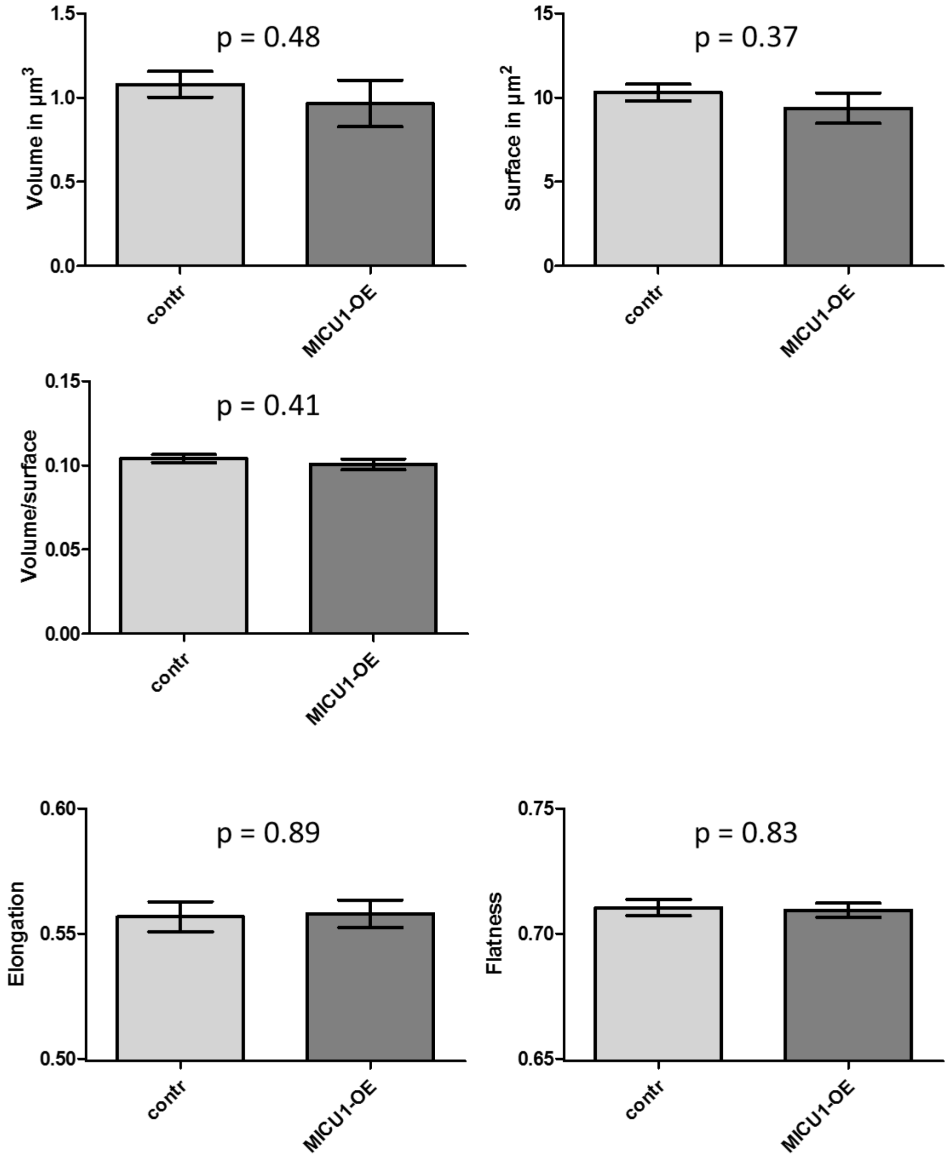

MICU1-YFP does not change mitochondrial morphology. HeLa cells transfected with and without MICU1-YFP were stained with MTR. Z-stacks were acquired, blind deconvoluted, background subtracted (rolling ball method, ImageJ) and analyzed for mitochondrial volume, surface, elongation and flatness. No significant changes of the mitochondrial morphology between MICU1-YFP and untransfected cells could be measured (n = 9). Data are shown as mean +/- SEM. P-values presented in the figure are calculated with unpaired double sided T-Test. Source data are provided as a Source Data file.

Supplementary Figure 2:

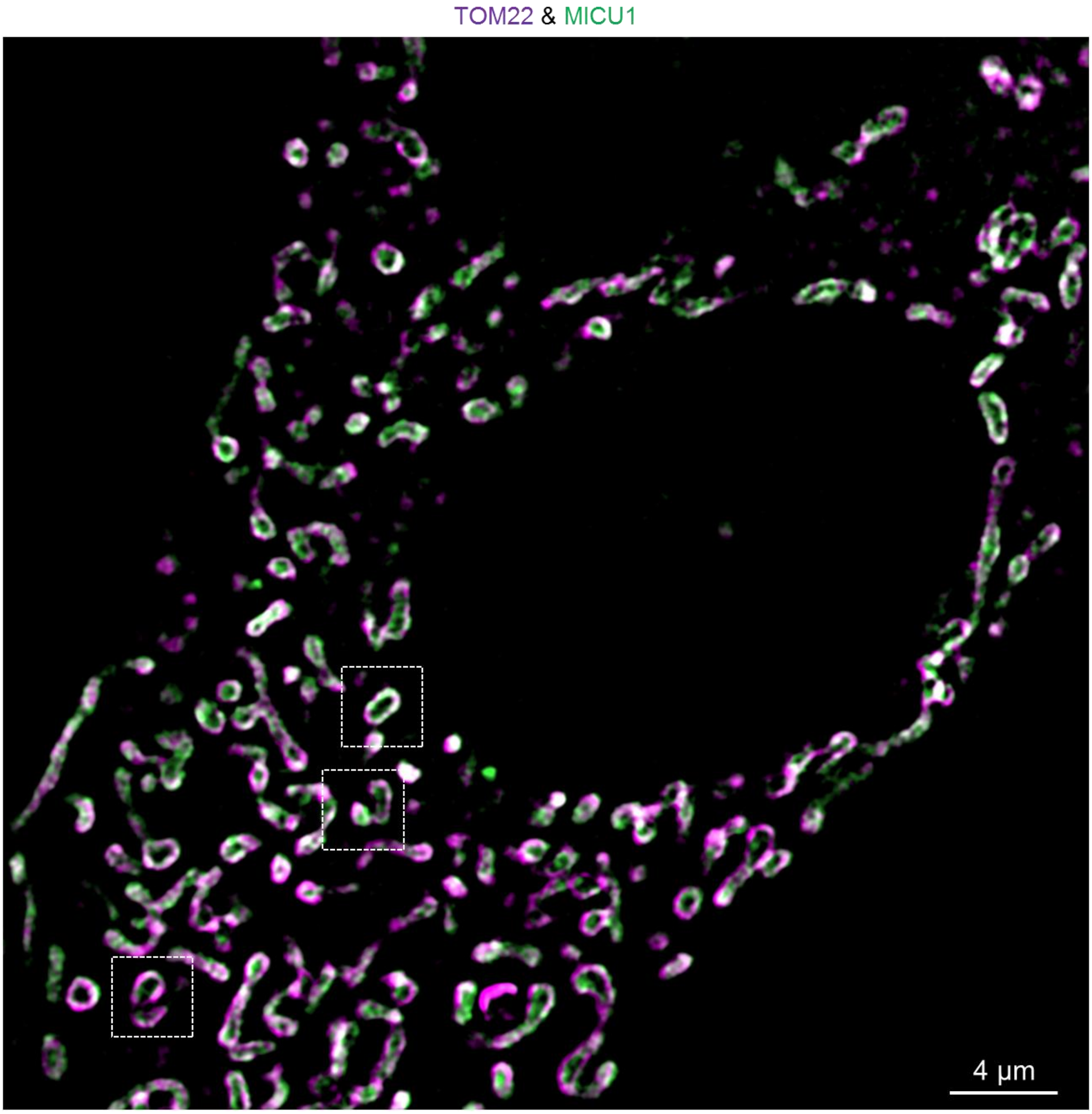

Overview of MICU1-YFP and mCherry-TOM22 transfected cell. HeLa cells were transfected with MICU1-YFP and mCherry-TOM22 constructs and imaged with Dual-SIM. White rectangles highlight the sections shown in Figure 1c.

Supplementary Figure 3:

A

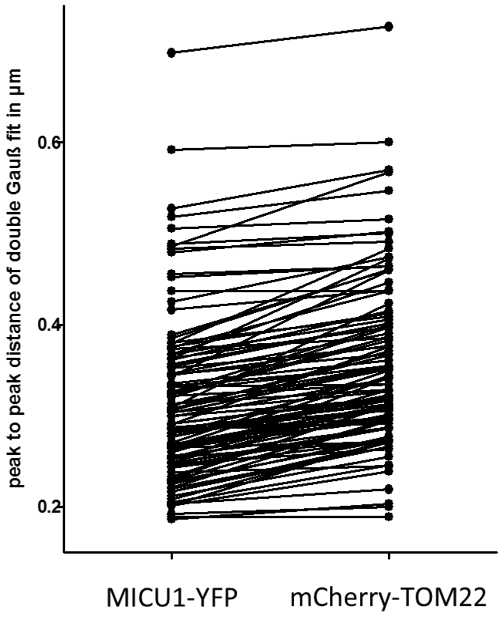

	MICU1-YFP	mCherry-Tom22
Number of values	105	105
Minimum	0.1863 µm	0.1892 µm
25% Percentile	0.2506 µm	0.2953 µm
Median	0.2991 µm	0.3407 µm
75% Percentile	0.3625 µm	0.4072 µm
Maximum	0.6982 µm	0.7271 µm
Mean	0.3171 µm	0.3596 µm
Std. Deviation	0.09390 µm	0.09390 µm
Std. Error	0.009163 µm	0.009164 µm
Lower 95% CI of mean	0.2990 µm	0.3414 µm
Upper 95% CI of mean	0.3353 µm	0.3777 µm

B

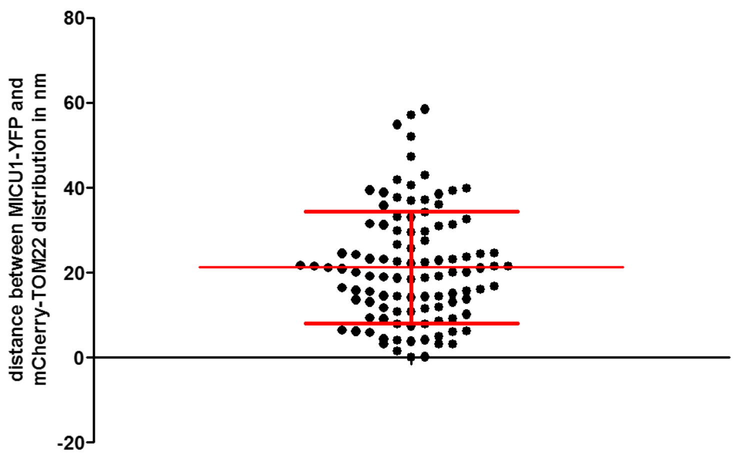

Number of values	105
Minimum	-0.01126 nm
25% Percentile	11.10 nm
Median	20.07 nm
75% Percentile	30.37 nm
Maximum	58.40 nm
Mean	21.21 nm
Std. Deviation	13.22 nm
Std. Error	1.290 nm
Lower 95% CI of mean	18.65 nm
Upper 95% CI of mean	23.76 nm

Detailed statistical analysis of MICU1-YFP and mCherry-TOM22 sub-mitochondrial distribution and spatial relation. HeLa cells were transfected with MICU1-YFP and mCherry-TOM22 constructs and imaged with Dual-SIM. (A) (*left panel*) Peak-to-peak distances of double Gaussian fit of MICU1 and TOM22 line plots were calculated and displayed as pairs for each mitochondrion. (*right panel*) Detailed statistical listing of MICU1-YFP and mCherry-TOM22 peak-to-peak distance distribution. (B) (*left panel*) The delta of peak-to-peak distances of MICU1 and TOM22 line plots were calculated. (*right panel*) Detailed statistical listing of the delta between MICU1-YFP and mCherry-TOM22 peak-to-peak distance distribution. Analyses were obtained from 4 - 5 mitochondria in at least 3 cells in each of 8 independent experiments. Data are shown as dot plots with the mean +/- SD as middle line and whiskers, respectively. (n = 105) Source data are provided as a Source Data file.

Supplementary Figure 4:

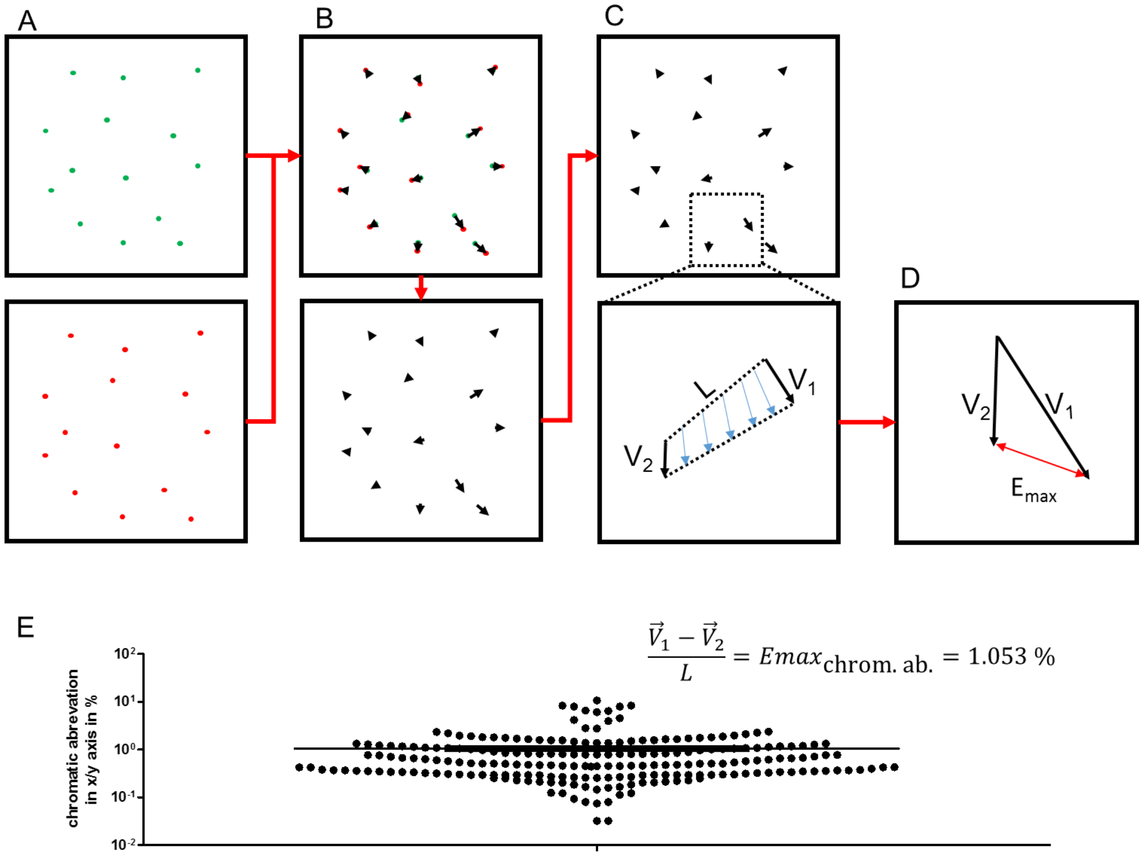

Schematic representation of Tetraspec bead measurements to identify the influence of chromatic abbreviations on the conducted N-SIM measurements. 100 nm Tetraspec beads were immobilized using poly-L-lysine on 1.5H high precision glass cover slips. (A) The Tetraspec beads (150 to 200 per image) were imaged using the Two-CAM system and illuminated simultaneously with 488 and 561 nm lasers. (B) Bead pairs in both channels were identified, localized with subpixel accuracy, and each pair got a vector assigned describing the local special chromatic abbreviation. (C) The closest neighbor of each bead was identified and the distance (L) was calculated using the green channel. (D) The vector difference of both beads was calculated and normalized to their distance. (E) A mean relative error of 1.053 % was calculated to be the systemic error introduced by chromatic abbreviations. Measuring a peak-to-peak distribution of MICU1-YFP or mCherry-TOM22 of 350 nm with the given setup would result in a systemic error introduction of approximately 3.5 nm. Data are shown as dot plots with the mean +/- SEM as middle line and whiskers, respectively (n = 210). Source data are provided as a Source Data file.

Supplementary Figure 5:

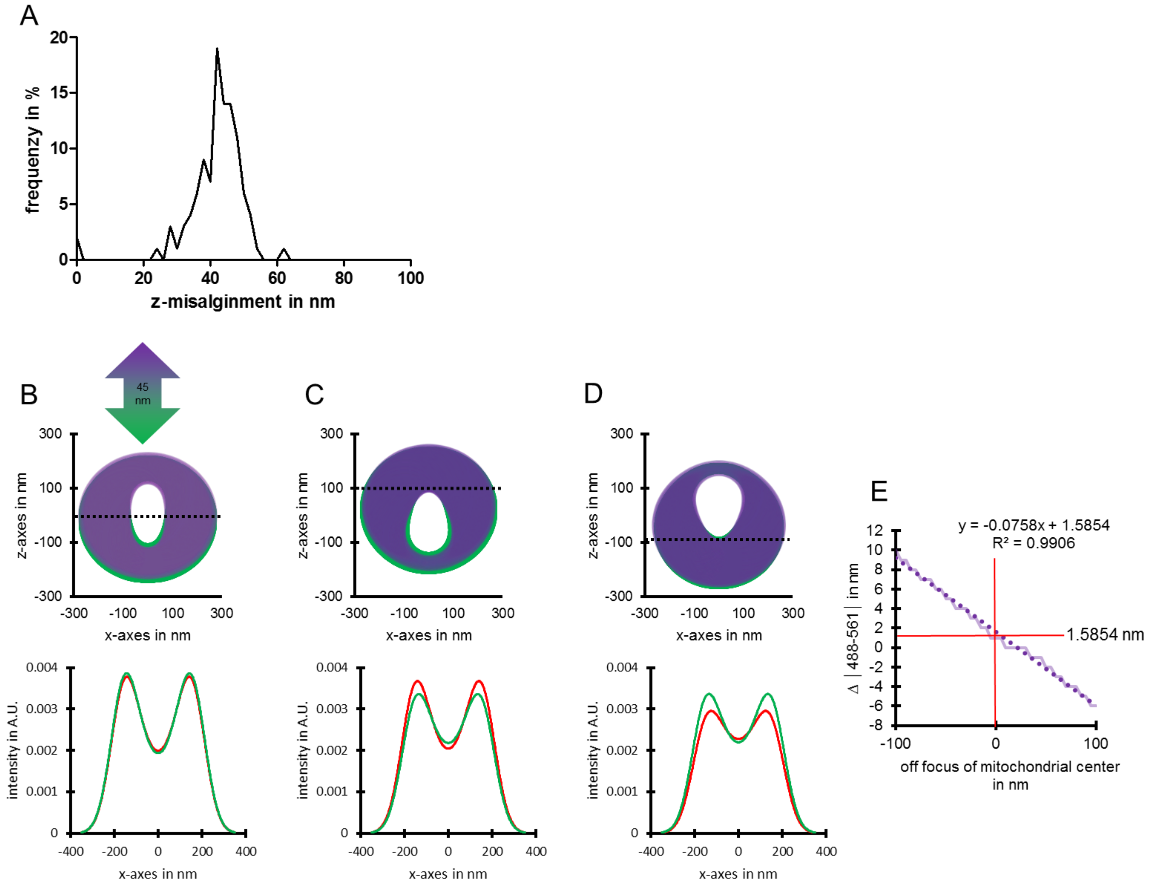

Measurement of chromatic abbreviations along the z-axes and analysis of the influence on peak to peak measurements. (A) Z-stacks of Tetraspec beads (150 to 200 per image) were acquired and the z-displacement between both channels was calculated by fitting the z-intensity projection to a Gaussian curve to determine the sub-pixel localization of the bead along the z-axes. The delta in position between both channels was plotted and the mean of 45 nm was used for further calculations. (B-D) Setting the 561 channel (magenta) as a reference and applying a point spread function with a delta along the z-axes of 45 nm to the 488 channel (green) results in a different fluorescence detection pattern, which in turn results in a change of the x-projection line plot and peak-to-peak distance. These changes in fluorescence detection, and peak-to-peak distance appear to be more severe if the focal plane is shifted 100 nm up or down. (E) Modeling the delta of peak-to-peak distances between the 488 and 561 channel dependent on the off focus in small increments of 5 nm and applying a linear fit leads to a good estimation of the error introduced by chromatic abbreviations along the z-axes. As the focus relative to the mitochondria can be estimated as normally distributed around the center, a standard error of approximately 1.6 nm can be applied. Source data are provided as a Source Data file.

Supplementary Figure 6:

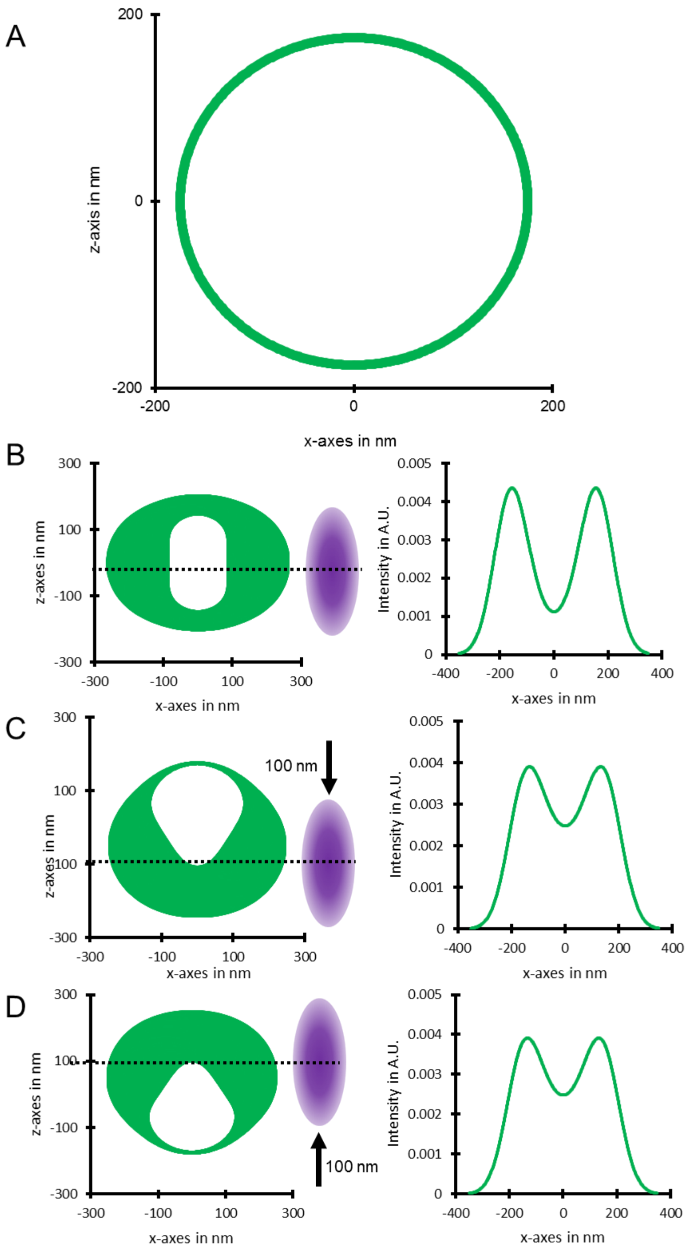

Model of the influence of the focus plane on mitochondrial line plots. (A) To evaluate if a different focus plane would change the line plot characteristics of mCherry-TOM22 or MICU1-YFP staining, we chose as a model of a hollow cylinder with its superficies fluorescently labeled. (B) The convolution of a the hollow cylinder model with the simplified point spread function (magenta ellipse; $FWHM_{x,y}$ = 120 nm; $FWHM_z$ = 260 nm) of the SIM-system in the center of the mitochondrion (black dotted line) yields a inhomogeneous but symmetrical fluorescence detection. That results in a x-projection showing a double peak structure. (C, D) Shifting the focus plane (black dotted line) 100 nm up or down results in an inhomogeneous and asymmetrical fluorescence detection and yields a narrower double peak structure of the x-projection line plot.

Supplementary Figure 7:

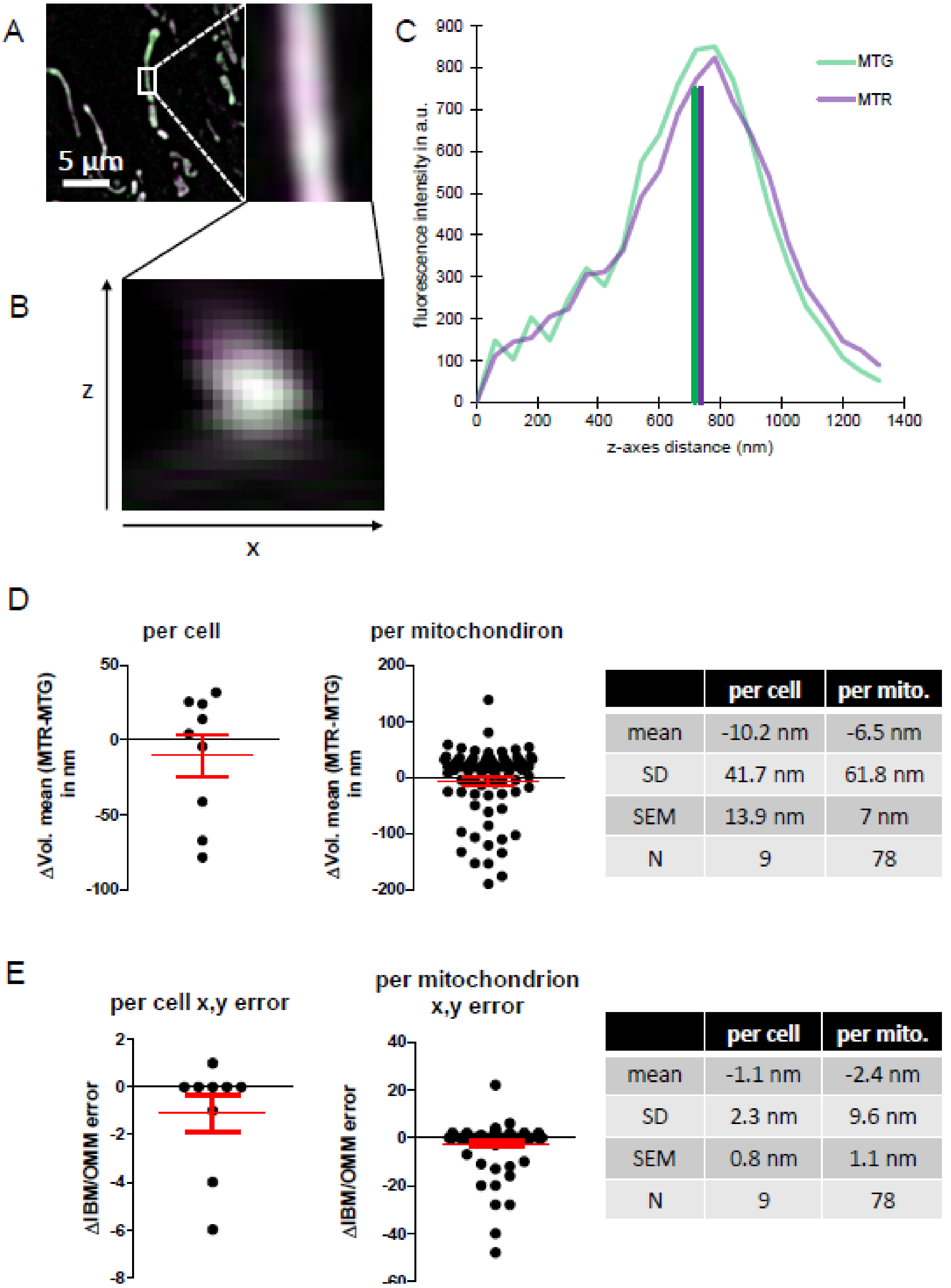

	per cell	per mito.
mean	-10.2 nm	-6.5 nm
SD	41.7 nm	61.8 nm
SEM	13.9 nm	7 nm
N	9	78

	per cell	per mito.
mean	-1.1 nm	-2.4 nm
SD	2.3 nm	9.6 nm
SEM	0.8 nm	1.1 nm
N	9	78

Analysis of z-misalignment induced by dispersion in live cells. (A) HeLa cells were grown on 1.5H glass cover slips and stained with MTG and MTR. Z-stacks were imaged in 60 nm increments using the dual-SIM system. (B) After background subtraction using the mosaic

suite tool single mitochondria were selected manually and transferred into z-projections. (C) The total fluorescence intensity of MTG and MTR was measured along the z-axes. The AUC (area under the curve) was measured and using linear interpolation for both labels the z-position was determined at which the AUC/2 is reached (straight lines). (D) The distance of the volumetric mean of both labels was set as the dispersion induced error in z. All values per cell and per mitochondrion are shown with the in red indicated mean and SEM. Mean, SD, SEM and the number of cell and mitochondria analyzed are listed. (E) Using the model in Supplementary Figure 5 & 6 the influence of the z-error was transformed into an estimation of the resulting error expected to see for TOM22 and MICU1 distance measurements. All values per cell and per mitochondrion are shown with the in red indicated mean and SEM. Mean, SD, SEM and the number of cell and mitochondria analyzed are listed. Data are shown as dot plots with the mean +/- SEM as middle line and whiskers, respectively. Source data are provided as a Source Data file.

Supplementary Figure 8:

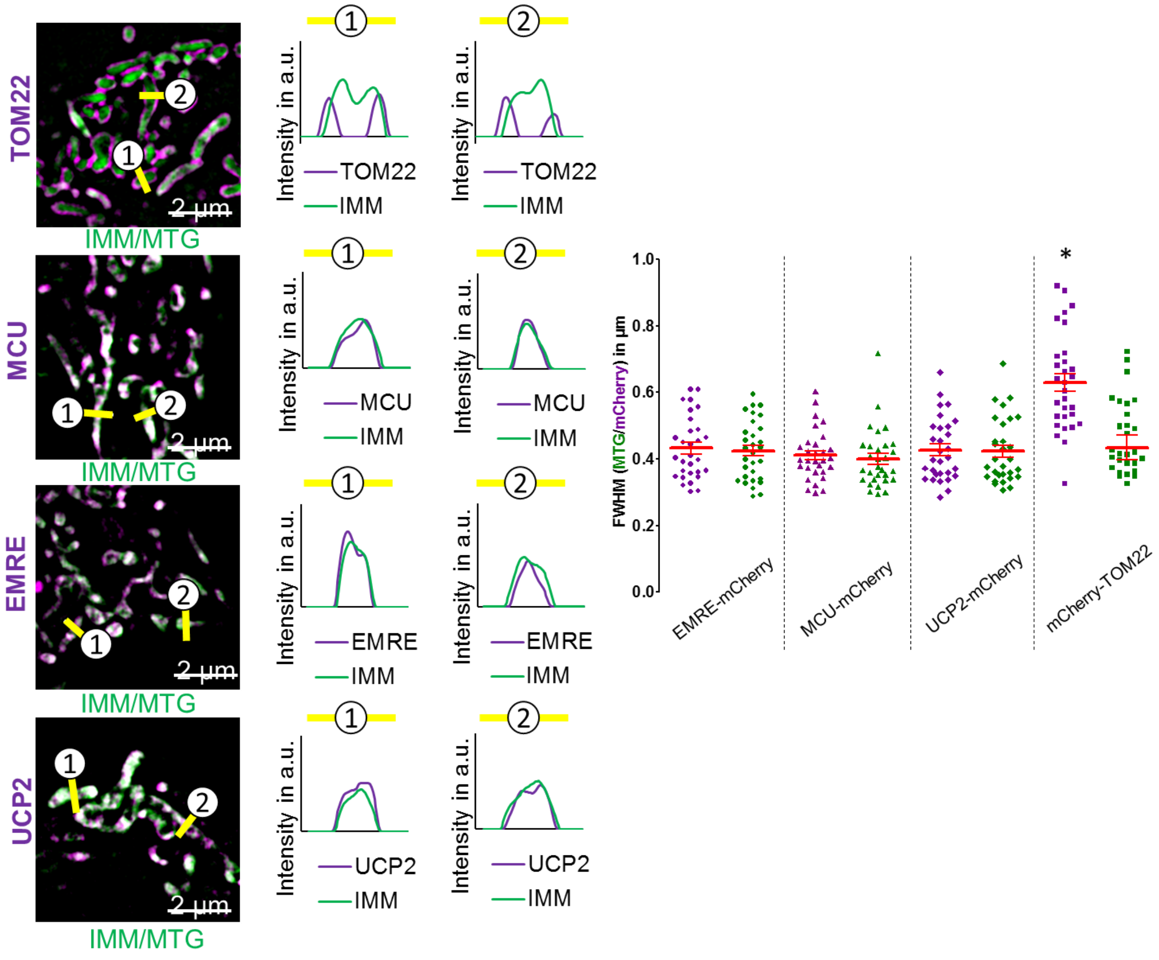

Representative images of mitochondrial proteins imaged with live 3D-SIM. HeLa cells were transiently transfected with either mCherry-TOM22 (TOM22), EMRE-mCherry (EMRE), MCU-mCherry (MCU) or UCP2-mCherry (UCP2) (all magenta), stained with Mitotracker Green® (MTG) (green) prior to experiments and imaged under resting conditions utilizing sequential 3D-SIM imaging. Excluding mCherry-TOM22, all images and line plots taken from the indicated dotted lines in the images revealed a ubiquitous distribution of these proteins over the entire IMM indicated by their co-localization with MTG. The mitochondrial FWHM was measured for MTG and mCherry signals in cells transfected with MCU-, EMRE-, or UCP2-mCherry or mCherry-TOM22 using lines plots with a width of 1 µm. No significant changes could be measured for the MTG FWHM while the mCherry-TOM22 FWHM was significantly increased compared to others. Data are shown as dot plots with the mean +/- SEM as middle line and whiskers, respectively (n = 30).* = P<0.05 vs. respective control conditions carried out

with analysis of variance (ANOVA) with Bonferroni post-hoc test.. Source data are provided as a Source Data file.

Supplementary Figure 9:

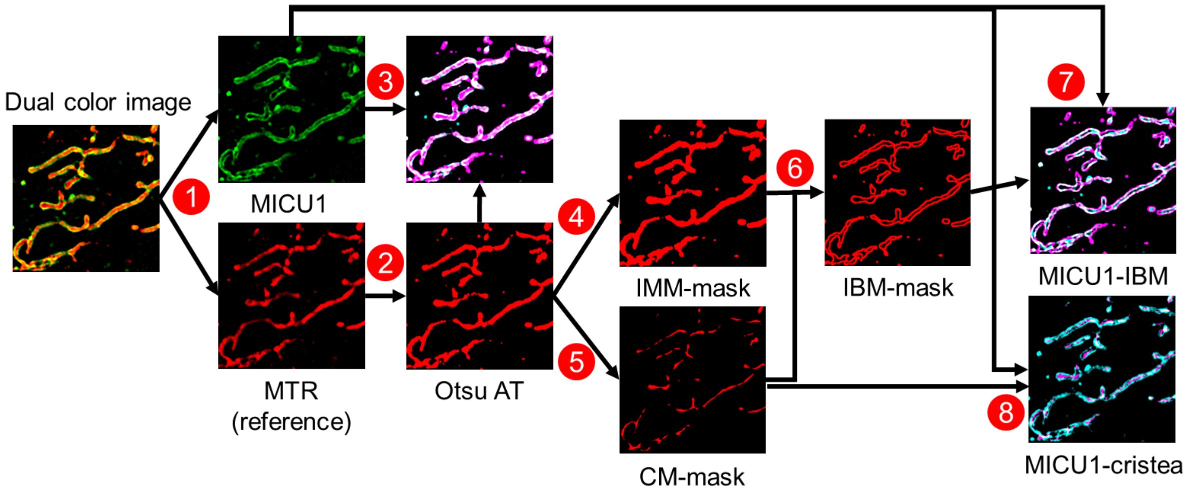

1. Splitting the image into the reference channel and the channel of interest
2. Otsu auto thresholding the reference channel
3. Overlay of thresholded mitochondrial reference with channel of interest
4. Dilation of the thresholded reference channel to generate the IMM-mask
5. Erosion of the thresholded reference channel to generate the CM-mask
6. Subtracting the CM-mask of the IMM-mask to generate the IBM-mask
7. Measuring the mean fluorescence intensity of the channel of interest in the IBM-mask
8. Measuring the mean fluorescence intensity of the channel of interest in the CM-mask
9. Calculating the IBM association index as the ratio of IBM-mask/CM-mask mean intensity

Image analysis scheme and description for the differentiation of the sub-mitochondrial protein localization. Dual color 3D-SIM images were split up into a reference and an object channel. The reference channel (MTR in this particular case) get Otzu auto thresholded followed by a dilation and erosion each. Subtracting the eroded images of the dilated gives a hollow tube binary mask. The mean fluorescence intensity measured within the hollow tube mask divided by the mean fluorescence intensity measured within the eroded binary mask yields a ratio expressing the sub-mitochondrial localization. The lower the ratio the higher the CM and lower the IBM localization. A high ratio states a high IBM and low CM protein distribution.

Supplementary Figure 10:

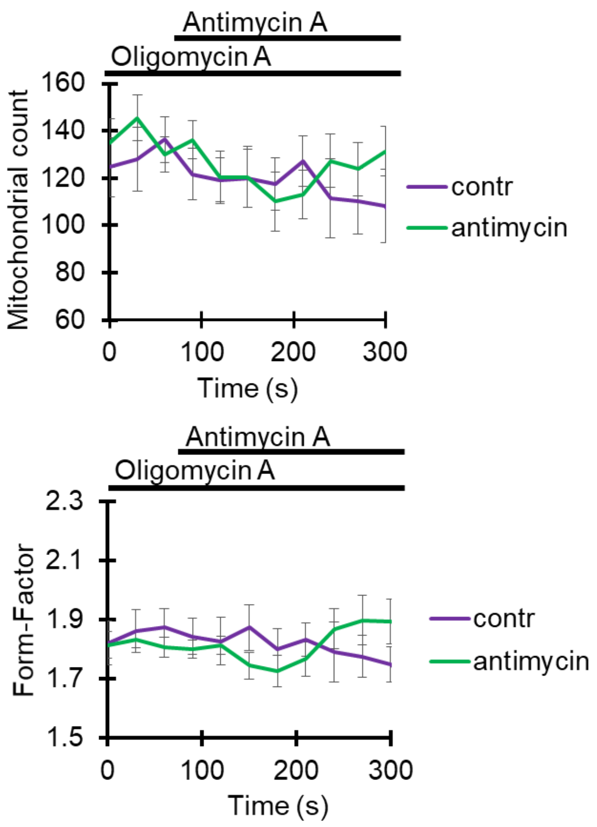

Antimycin A and oligomycin A treatments do not affect mitochondrial morphology. The EMRE-mCherry fluorescence was used to confirm the mitochondrial integrity over time. None of the observed characteristics was changed significantly after Antimycin A addition over time ($n_{Antimycin}$ = 8; $n_{Antimycin/Oligomycin}$ = 9). Data are shown as mean +/- SEM. * = *P<0.05* vs. respective control conditions calculated with unpaired double sided T-Test. Source data are provided as a Source Data file.

Supplementary Figure 11:

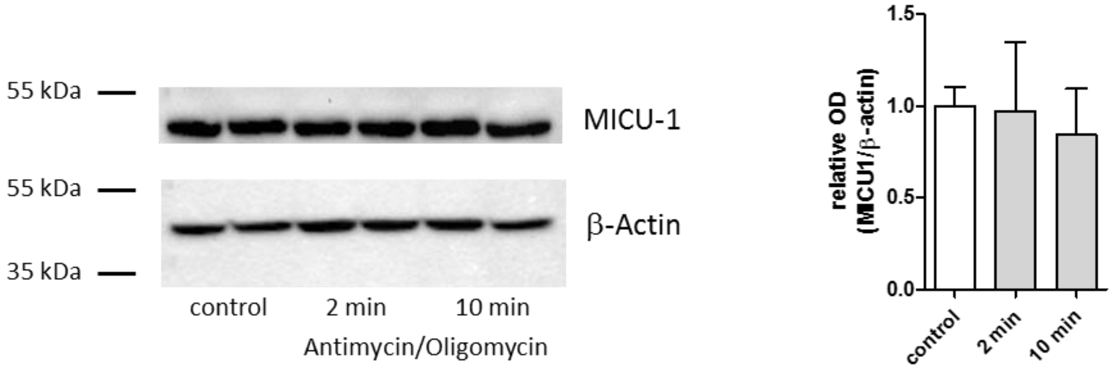

MICU1 does not undergo proteolytic cleavage during antimycin A and oligomycin A treatment. HeLa cells transfected with MICU1-YFP were treated for 2 or 10 min with antimycin A/oligomycin A, lysed and analyzed for MICU1 using Western blotting. No antimycin A/oligomycin A related degradation was observed (n = 4). Data are shown as mean +/- SD. * = P<0.05 vs. respective control conditions carried out with analysis of variance (ANOVA) with Bonferroni post-hoc test. Source data are provided as a Source Data file.

Supplementary Figure 12:

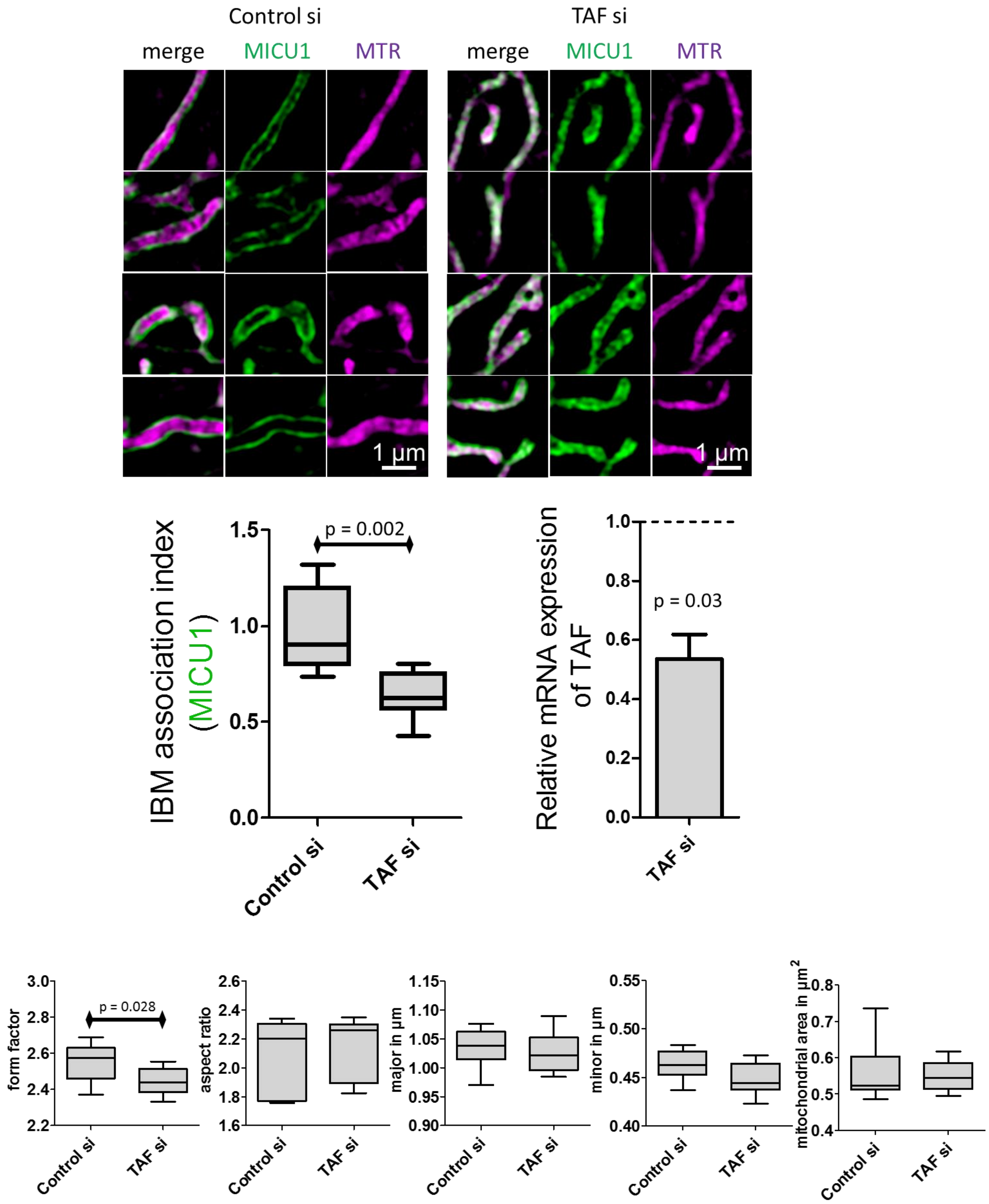

Knock-down of taffazin (TAF) leads to reorganization of MICU1 into the whole IMM. HeLa cells transfected with MICU1-YFP and control or TAF siRNA were imaged using structured illumination microscopy. Representative images of each 4 different cells transfected with control or TAF siRNA are shown. The IBM association factor as well as mitochondrial morphology were extracted from the images (n = 9). To verify the knockdown efficacy qRT-PCR was conducted for control and TAF (taffazin) siRNA transfected cells (n = 3). Horizontal lines represent the median, the lower and upper hinge show respectively first quartile and third quartile, and lower and upper whisker encompass minimal and maximal values. P-values

presented in the figure are calculated with unpaired double sided T-Test. Source data are provided as a Source Data file.

Supplementary Figure 13:

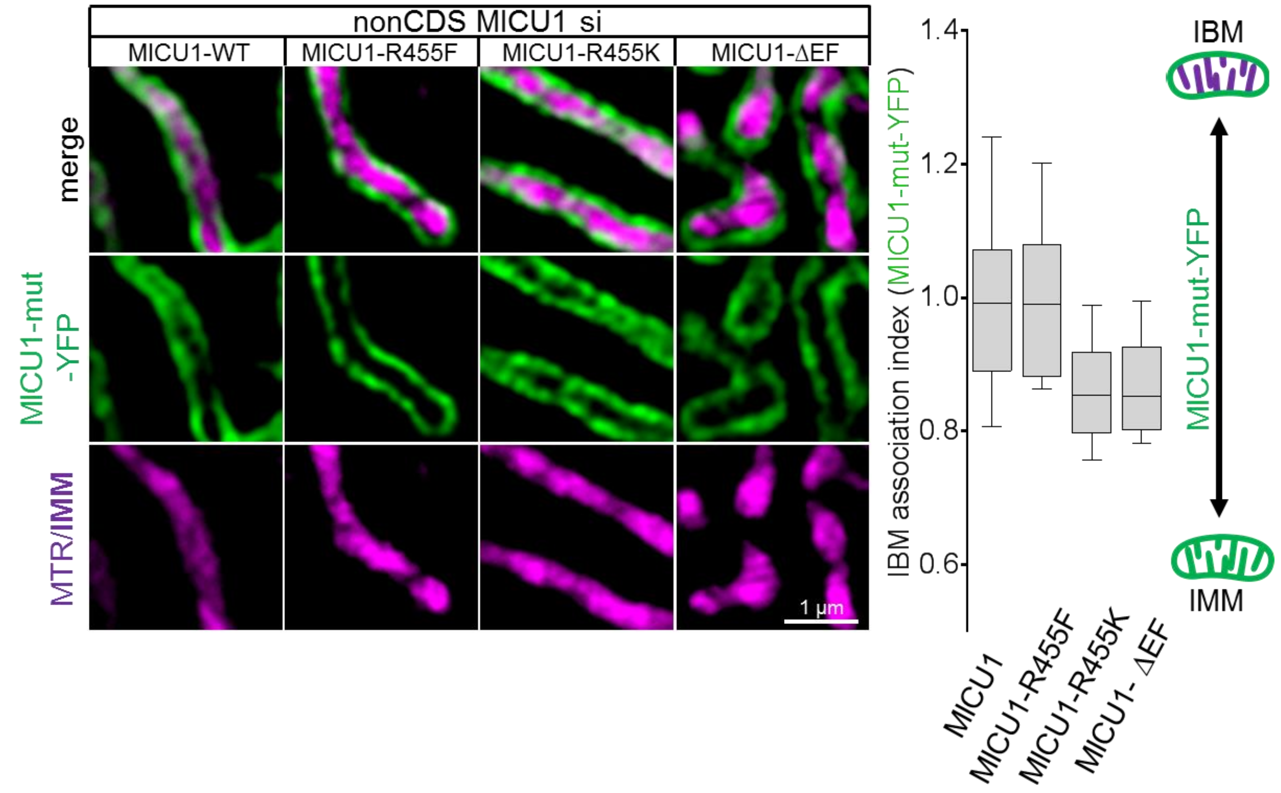

MICU1-F, MICU1-K, and MICU1-EF mutants have the same IBM localization like the wildtyp MICU1. HeLa cells were transfected with MICU1-WT or MICU1-F, MICU1-K, or MICU1-EF mutants tagged to YFP and stained for 40 min in loading buffer with 0.5 µM MTR. The IBM association index was determined for the MICU1 wildtype and mutants using the MTR/IMM staining as reference. (n_{MICU1} = 13; $n_{MICU1\text{-}K455F}$ = 6; $n_{MICU1\text{-}K455K}$ = 8; $n_{MICU1\text{-}\Delta EF}$ = 6). Horizontal lines represent the median, the lower and upper hinge show respectively first quartile and third quartile, and lower and upper whisker encompass minimal and maximal values. *P<0.05 vs. respective control conditions carried out with analysis of variance (ANOVA) with Bonferroni post-hoc test. Source data are provided as a Source Data file.

Supplementary Figure 14:

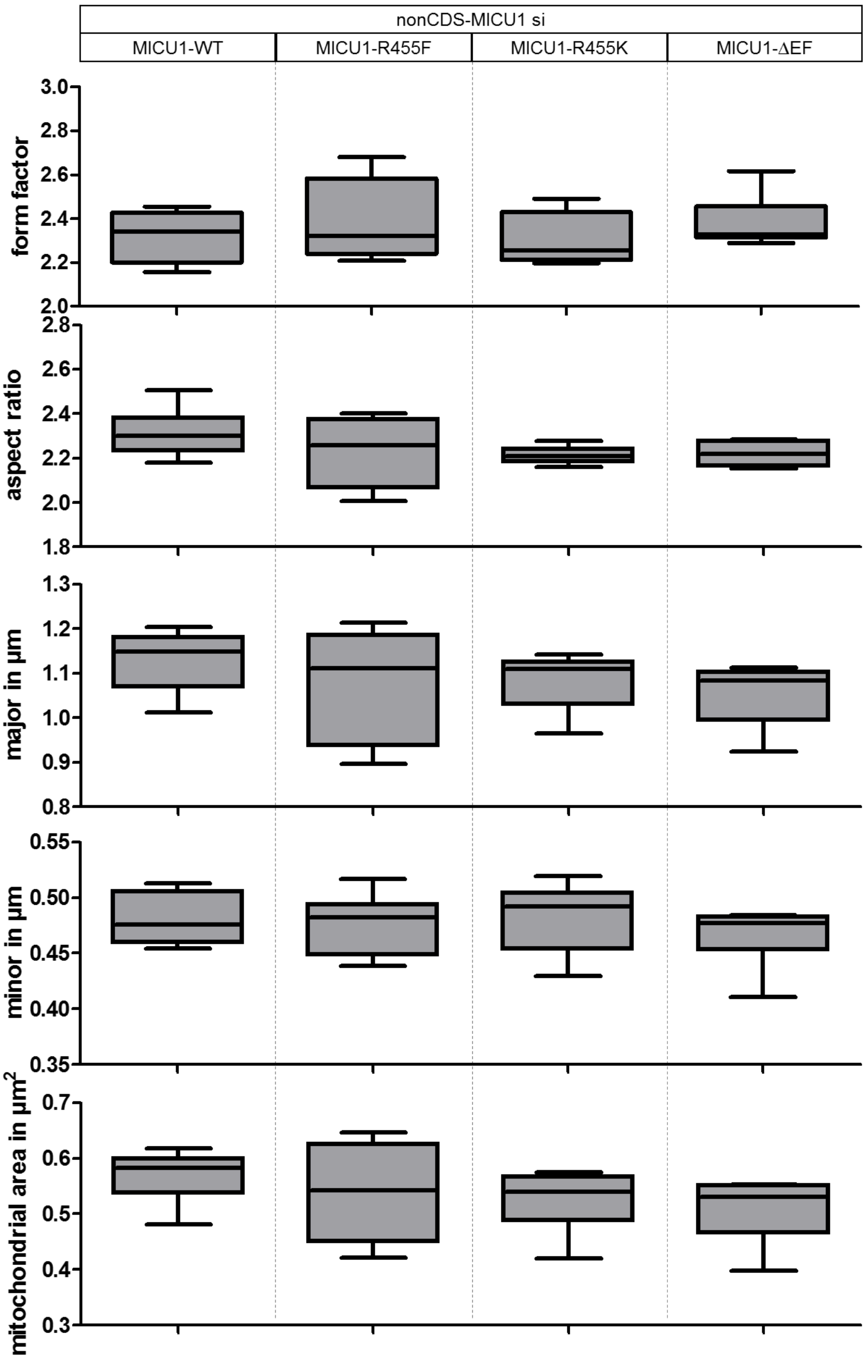

MICU1-F, MICU1-K, and MICU1-EF mutant-expressing HeLa cells have the same mitochondrial morphology as wildtype MICU1-expressing cells. HeLa cells were transfected with MICU1-WT or MICU1-F, MICU1-K, or MICU1-EF mutants tagged to YFP and stained for 40 min in loading buffer with 0.5 µM MTR. Form factor, aspect ratio, major/minor diameters and mitochondrial area were determined for the MICU1 wildtype and mutants using the MTR/IMM staining (n_{MICU1} = 13; $n_{MICU1\text{-}K455F}$ = 6; $n_{MICU1\text{-}K455K}$ = 8; $n_{MICU1\text{-}\Delta EF}$ = 6). Horizontal lines represent the median, the lower and upper hinge show respectively first quartile and third quartile, and lower and upper whisker encompass minimal and maximal values. *P<0.05 vs.

respective control conditions carried out with analysis of variance (ANOVA) with Bonferroni post-hoc test. Source data are provided as a Source Data file.

Supplementary Figure 15:

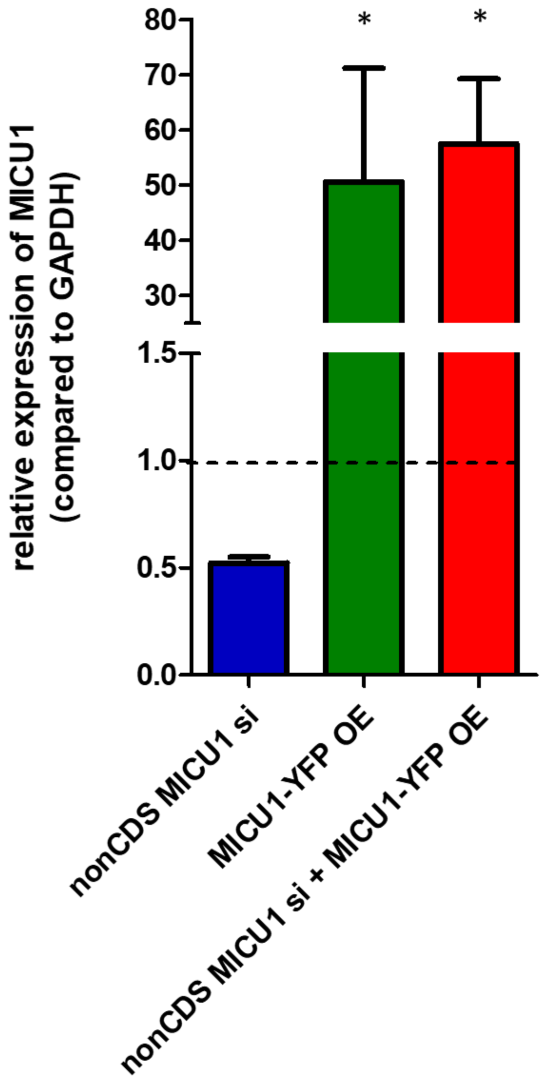

Knock down efficiency and specificity of nonCDS MICU1si. HeLa cells were transfected with MICU1-YFP (OE) and control or nonCDS MICU1 siRNA. qRT-PCR was performed to show knockdown efficiency and specificity of endogenous MICU1. Analyses were obtained from 3 different experiments on 3 independent days (n = 3). Data are shown as mean +/- SEM. *$P<0.05$ vs. respective control conditions carried out with analysis of variance (ANOVA) with Bonferroni post-hoc test. Source data are provided as a Source Data file.

Supplementary Figure 16:

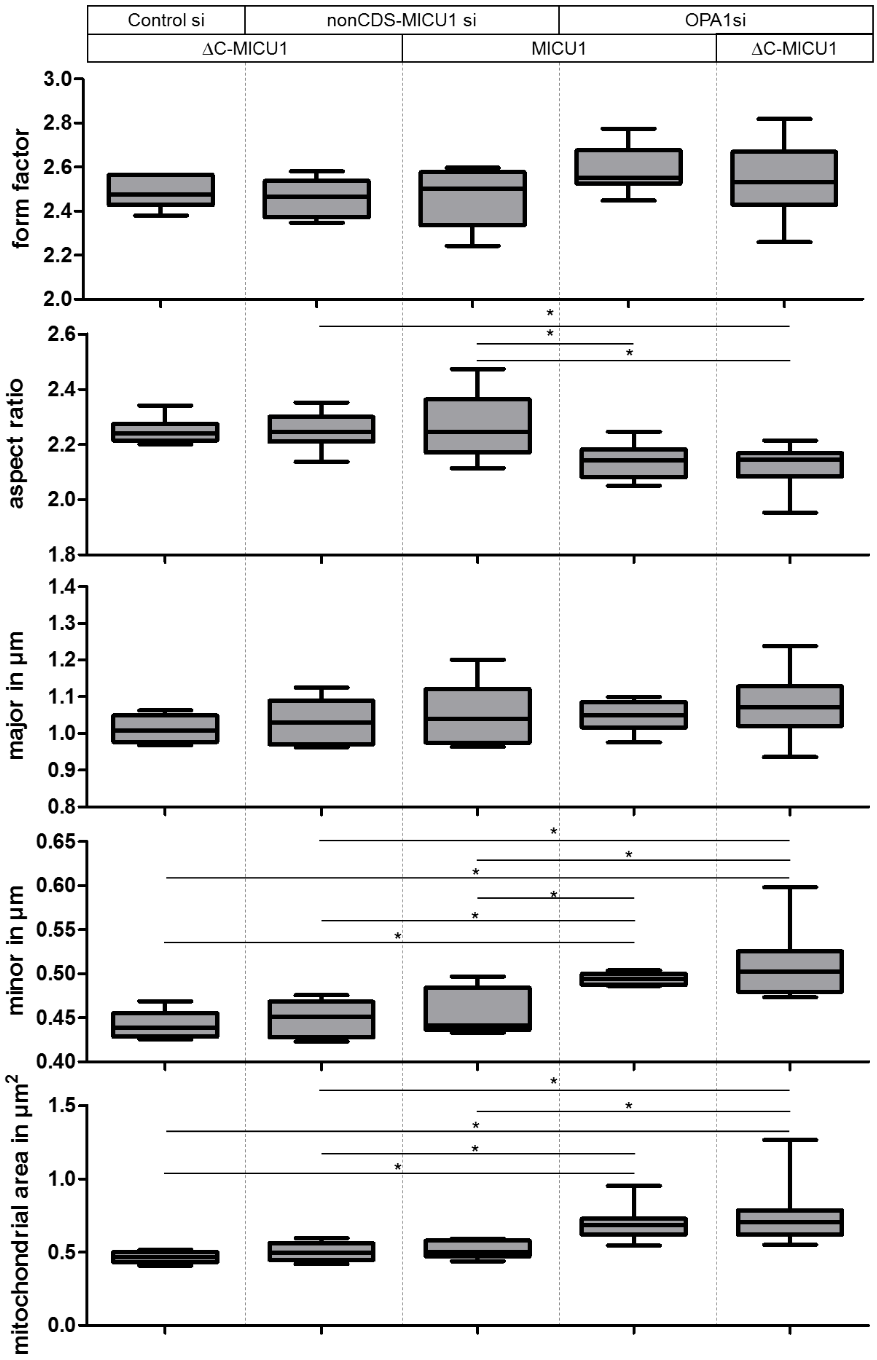

Influence of OPA1 and endogenous MICU1 silencing on mitochondrial morphology. HeLa cells stained with MTR (magenta) and expressing ΔC-MICU1-YFP or MICU1-YFP and transfected with either non-coding siRNA against MICU1 (nonCDS-MICU1 si), OPA1 (OPA1 si) or control siRNA (Control si). Form factor, aspect ratio, major/minor diameters, and mitochondrial area were determined for the MICU1 wildtype and mutants using the MTR/IMM staining (n = 8). Horizontal lines represent the median, the lower and upper hinge show respectively first quartile and third quartile, and lower and upper whisker encompass minimal and maximal values. *P<0.05 vs. respective control conditions carried out with analysis of

variance (ANOVA) with Bonferroni post-hoc test. Source data are provided as a Source Data file.

Supplementary Figure 17:

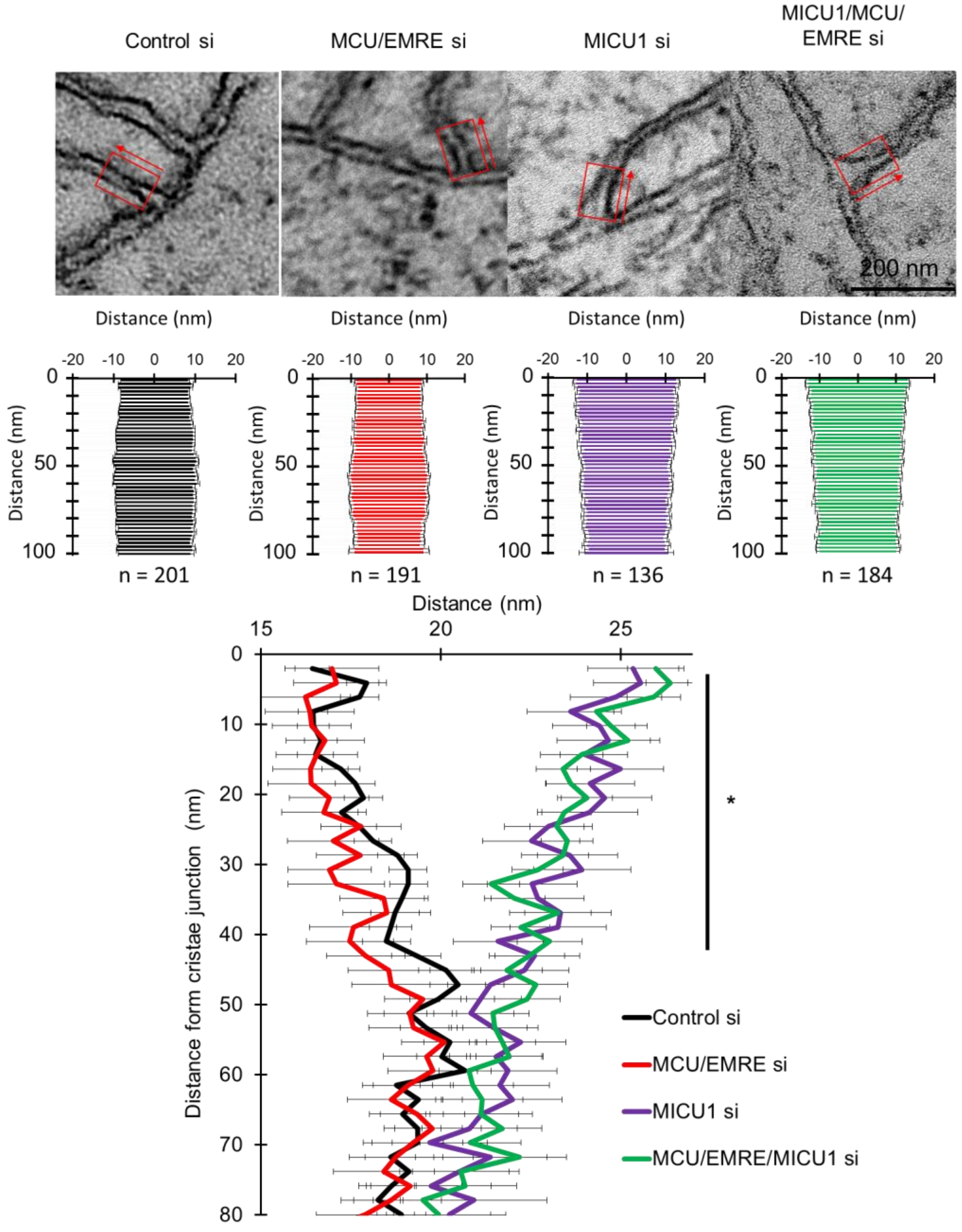

MCU/EMRE silencing does not influence the CJ width. HeLa cells transfected with control (n = 3/21/201 with preparations/cells/CJ), MICU1 (n = 3/20/136 with preparations/cells/CJ), MCU/EMRE (n = 3/22/191 with preparations/cells/CJ) or MICU1/MCU/EMRE (n = 3/20/184 with preparations/cells/CJ) siRNA were imaged with transmission electron microscopy. To analyze the topology of the cristae junction the cristae width was measured in 2 nm increments starting at the perceived protrusion of the IBM and measured into the cristae, as indicated with red boxes and arrows in the upper panel. The comparative analysis shows no influence of MCU/EMRE silencing on the CJ width while MICU1 knockdown increases the CJ width significantly for the first 40 nm of the membrane invagination. Data are shown as mean +/-

SEM ($n_{Control\ si}$ = 201; $n_{MCU/EMRE\ si}$ = 191; $n_{MICU1\ si}$ = 136; $n_{MCU/EMRE/MICU1\ si}$ = 184). *$P<0.05$ vs. respective control conditions carried out with analysis of variance (ANOVA) with Bonferroni post-hoc test. Source data are provided as a Source Data file.

Supplementary Figure 18:

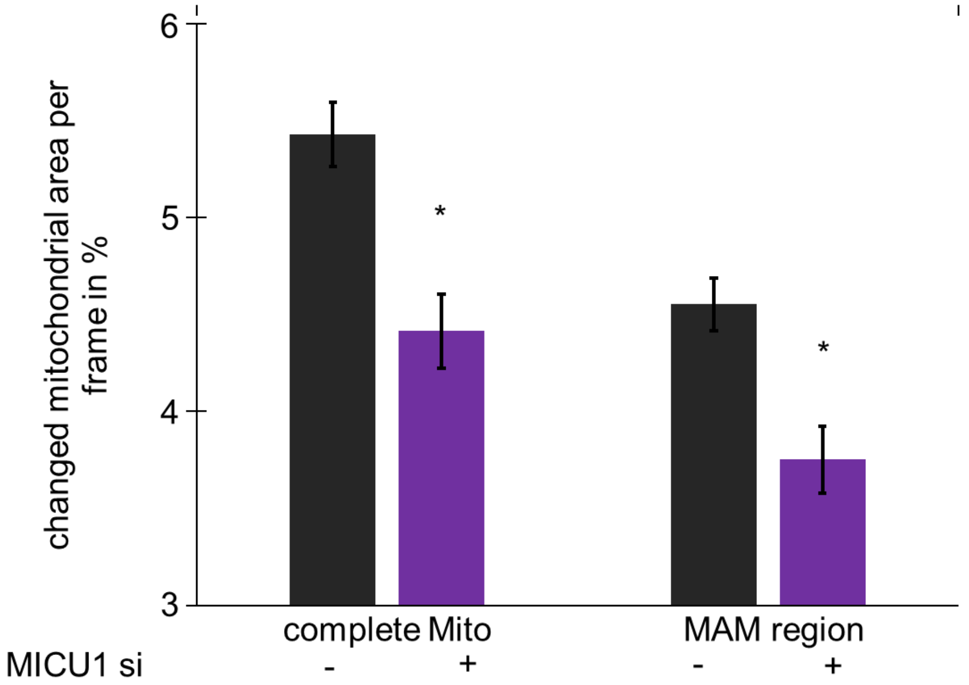

MICU1 knockdown is decreasing inner mitochondrial membrane kinetics in whole mitochondria as well in close proximity to the endoplasmic reticulum. HeLa cells were transfected with ER-RFP and control or MICU1 siRNA and stained with 0.5 µM MTG for 40 min. The CM-dynamics were quantified with the whole mitochondria as well as in mitochondrial areas with a close proximity to the ER. Images and analyses were obtained from n = 8 independent experiments, which assessed a total of 40 cells. Data are shown as mean +/- SEM. * = $P<0.05$ vs. respective control conditions calculated with unpaired double sided T-Test. Source data are provided as a Source Data file.

Supplementary Figure 19:

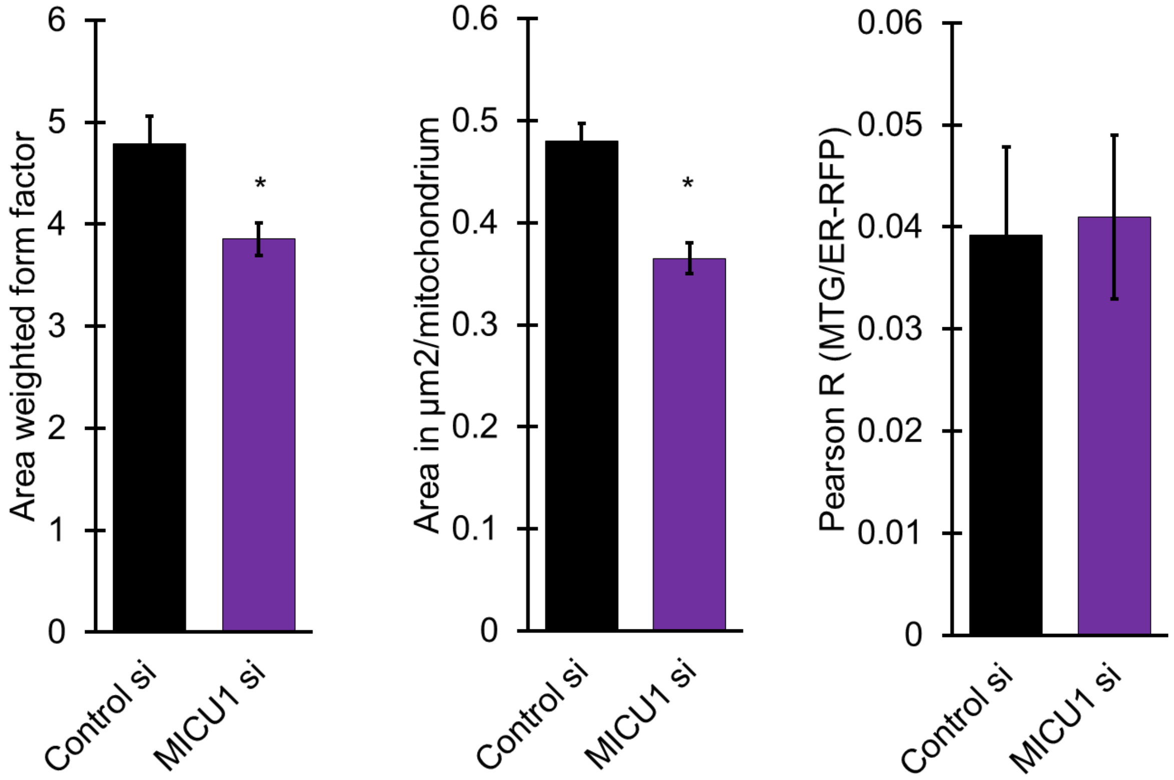

MICU1 knockdown is decreasing mitochondrial branching but MAMs are not affected. HeLa cells were transfected with ER-RFP and control or MICU1 siRNA and stained with 0.5 µM MTG for 40 min. the area weighted form factor, mean mitochondrial area and the co-localization of MTG and ER-RFP as a measure of MAMs were determined using Fiji. Images and analyses were obtained from n = 8 independent experiments, which assessed a total of 40 cells. Data are shown as mean +/- SEM. * = $P<0.05$ vs. respective control conditions calculated with unpaired double sided T-Test. Source data are provided as a Source Data file.

Supplementary Figure 20:

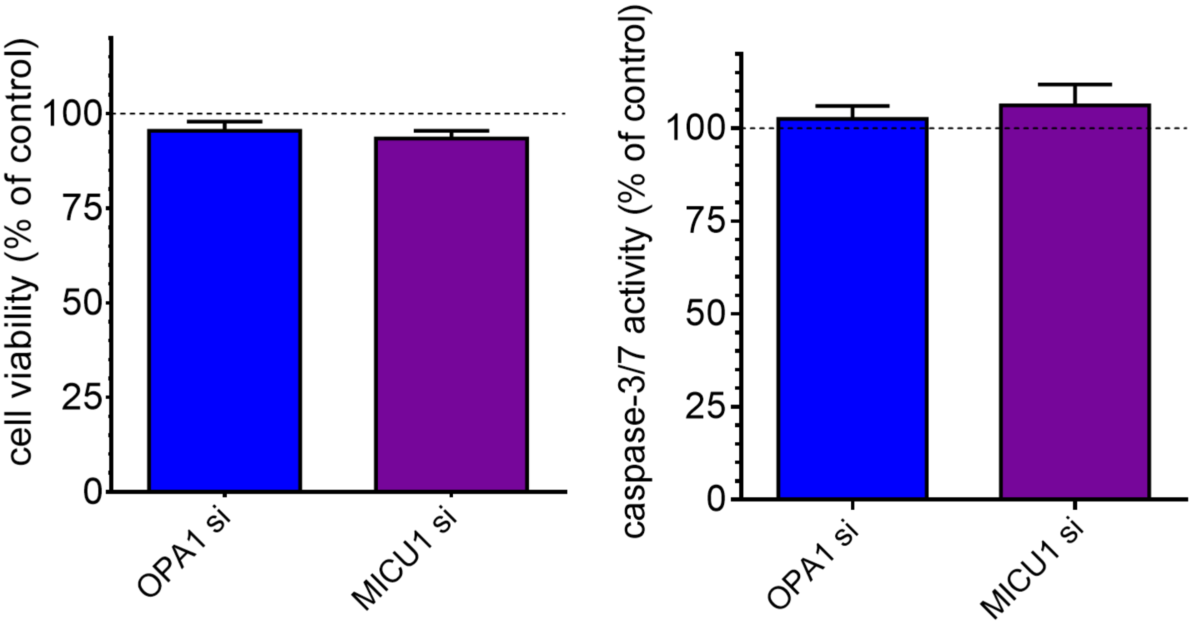

Knock down of OPA1 or MICU1 in HeLa cells is not changing the cell viability and caspase-3/7 activity. HeLa cell were transfected with control, MICU1, or OPA1 siRNA. Cell viability and apoptotic levels were measured with CellTiter-Blue assay (Promega) and Caspase-Glo®-3/7 assay (Promega) respectively. No significant changes for cell viability or caspase activity could be determined. Analyses were obtained from 5 different experiments on 2 independent days (n = 5). Data are shown as mean +/- SEM. * = *P<0.05* vs. respective control conditions calculated with unpaired double sided T-Test. Source data are provided as a Source Data file.

Supplementary Figure 21:

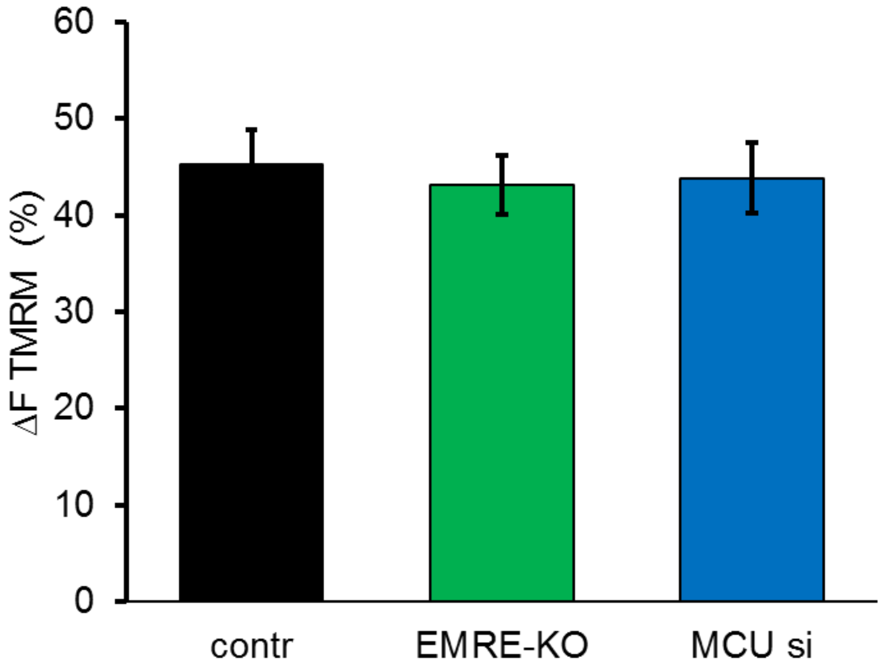

Silencing of MCU or knockout of EMRE does not affect mitochondrial membrane potential. HeLa cell transfected with control or MCU siRNA and HeLa-EMRE-KO cells were stained with TMRM. The TMRM Δ fluorescence intensity after addition of 2 µM FCCP was measured (n = 9). Data are shown as mean +/- SEM. * = *P<0.05* vs. respective control conditions calculated with analysis of variance (ANOVA) with Bonferroni post-hoc test. Source data are provided as a Source Data file.

Supplementary Figure 22:

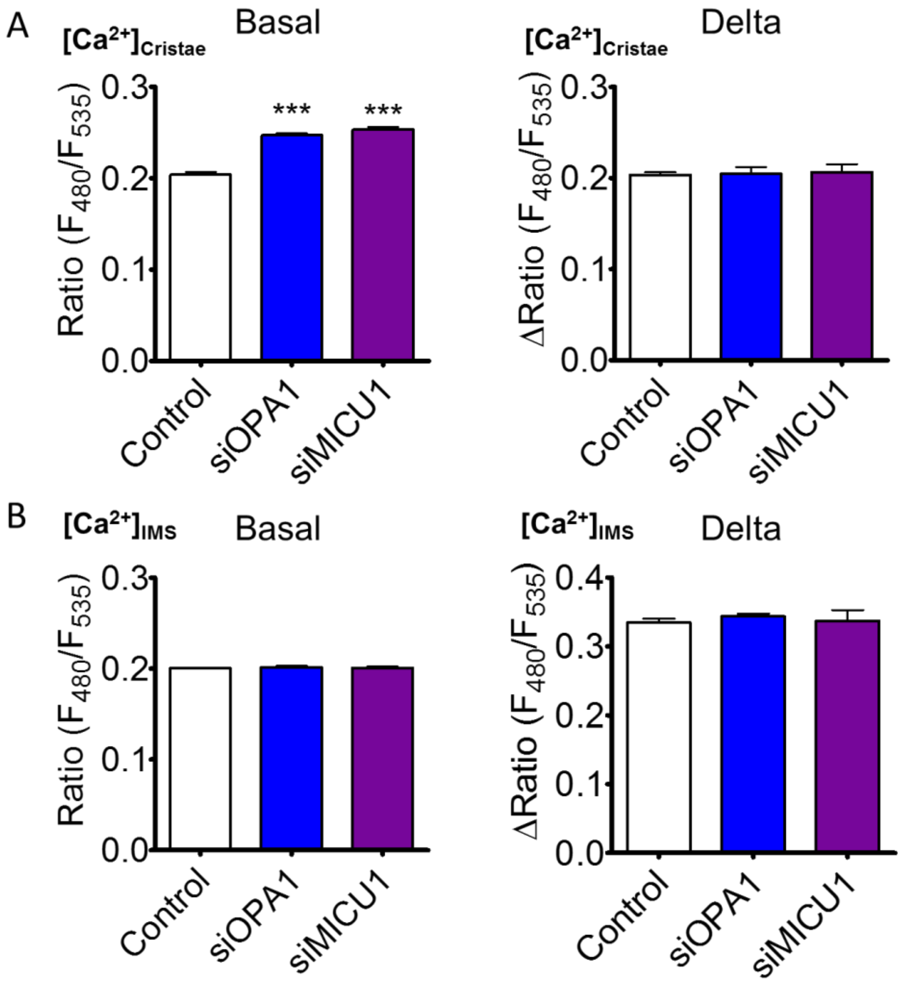

Statistical analysis of cristae and IMS Ca^{2+} traces. (A) Bars show basal values (left panel) and maximal Δ ratio values (right panel) in response to 100 µM histamine in Ca^{2+}-free conditions of CL-GEMGeCO1 expressing HeLa cells treated with control, OPA1, or MICU1 siRNA ($n_{Control\ si}$ = 9; $n_{OPA1\ si}$ = 10; $n_{MICU1\ si}$ = 10). (B) Bars show basal values (left panel) and maximal Δ ratio values (right panel) in response to 100 µM histamine in Ca^{2+}-free conditions of IMS-GEMGeCO1 expressing HeLa cells treated with control, OPA1, or MICU1 siRNA ($n_{Control\ si}$ = 8; $n_{OPA1\ si}$ = 11; $n_{MICU1\ si}$ = 7). Data are shown as mean +/- SEM. * = *P<0.05* vs. respective control conditions carried out with analysis of variance (ANOVA) with Bonferroni post-hoc test. Source data are provided as a Source Data file.

Supplementary Figure 23:

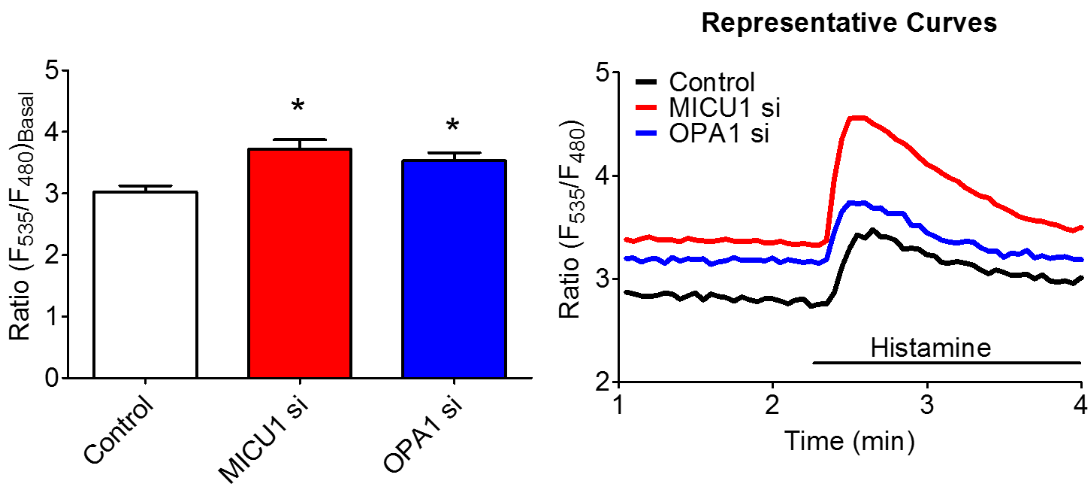

Knock down of OPA1 or MICU1 is increasing basal mitochondrial matrix Ca^{2+} HeLa cells transfected with siRNA against OPA1 or MICU1 or scrambled siRNA expressing the mitochondrial matrix Ca^{2+} sensor 4mt3cpv were challenged with 100 µM histamine. The basal Ca^{2+} concentration for both OPA1 and MICU1 knockdown was significantly increased compared to control condition ($n_{Control\ si}$ = 18; $n_{OPA1\ si}$ = 18; $n_{MICU1\ si}$ = 16). Data are shown as mean +/- SEM. * = *P<0.05* vs. respective control conditions carried out with analysis of variance (ANOVA) with Bonferroni post-hoc test. Source data are provided as a Source Data file.

Supplementary Figure 24:

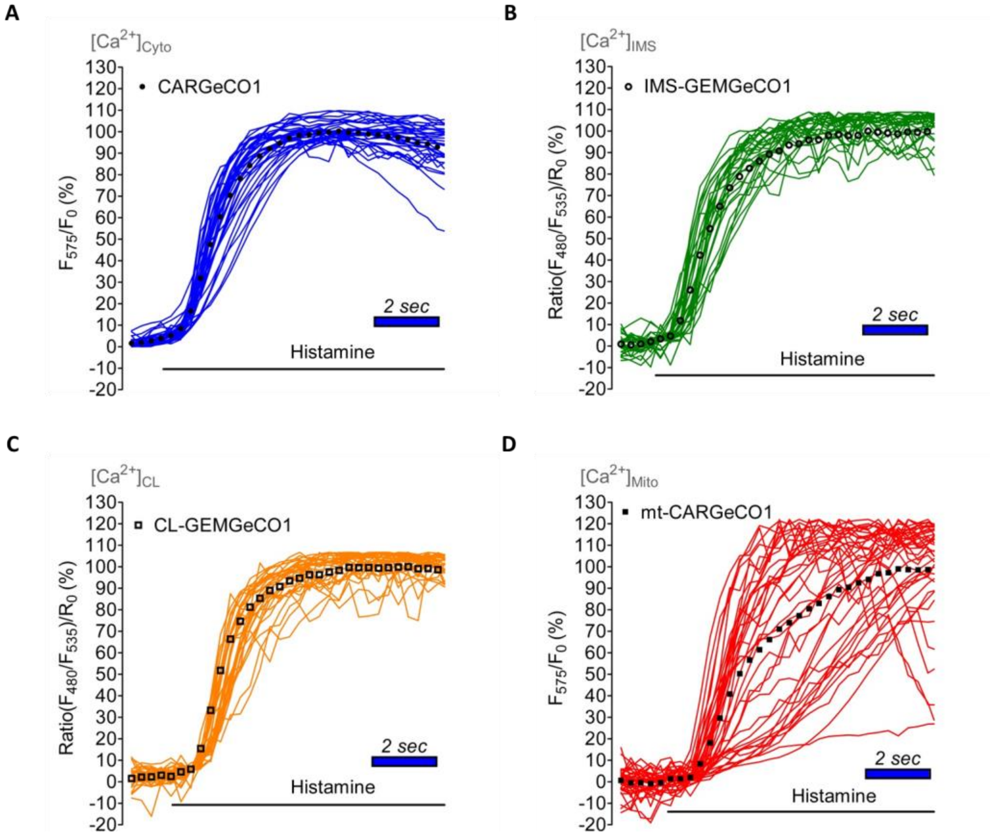

Dynamics of HeLa cell responses to histamine. Cells co-expressing red and green localized GeCO1 indicators were simultaneously measured within a total cycle time of 300 ms upon histamine stimulation in a Ca^{2+}-free environment. Individual cell responses were normalized to the maximum value of the average curves to 100 % for either (A) cytosolic (individual blue curves with filled black circles indicating the average curve, (B) IMS (dark green curves with clear black circles), (C) CL (light orange curves with filled black squares) or (D) mitochondrial matrix Ca^{2+} rises (red curves with clear black squares). Source data are provided as a Source Data file.

Supplementary Figure 25:

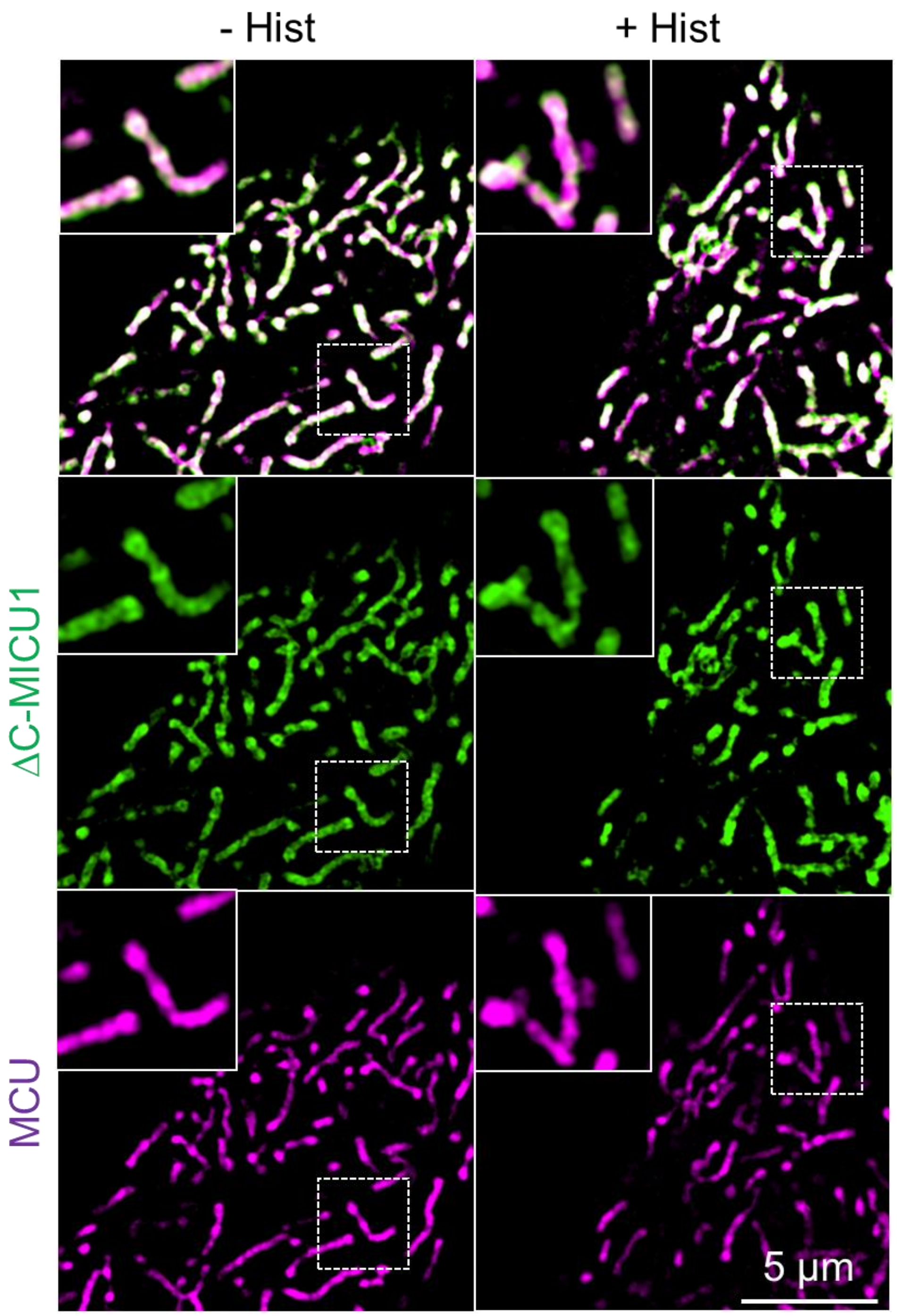

The ΔC-MICU1-YFP mutant does not recruit MCU-mCherry into the IBM in response to histamine treatment. Representative images of HeLa cells transfected with MCU-mCherry and ΔC-MICU1-YFP imaged with and without the presence of histamine. Images were obtained from at least 10 cells in each of 2 different experiments on 4 independent days (n=8). **P<0.01* vs. respective control conditions.

Supplementary Figure 26:

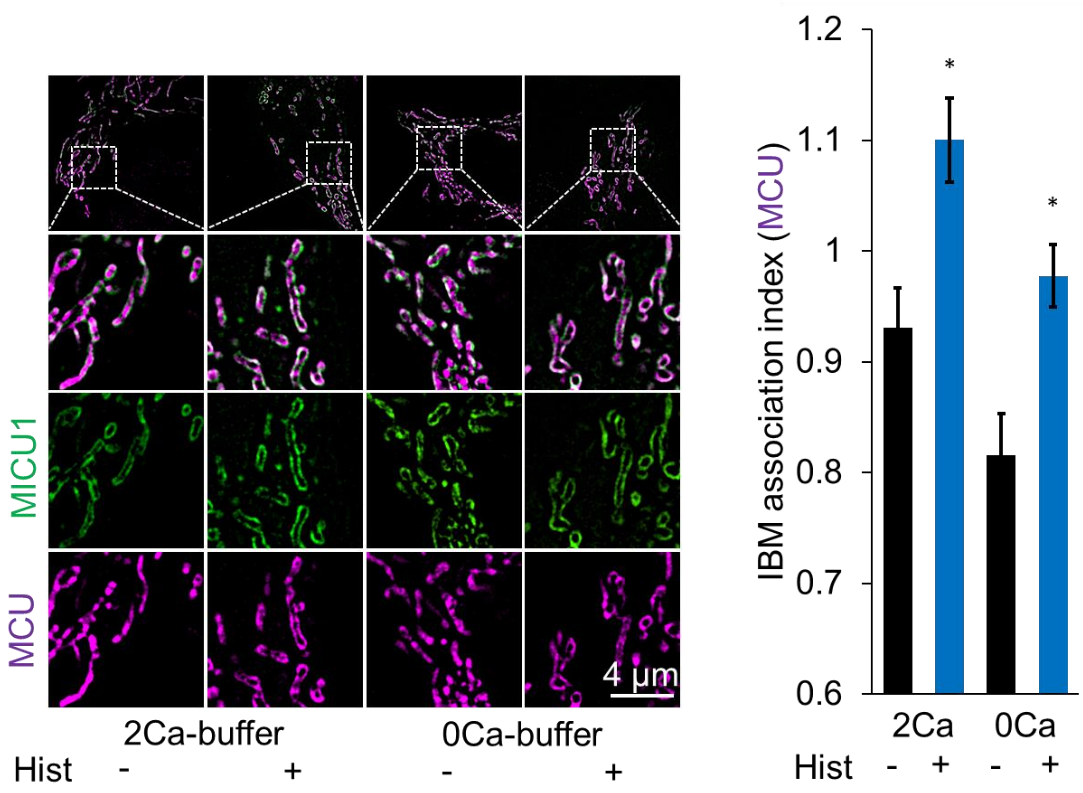

MCU shuttling is independent of extracellular Ca^{2+}. HeLa cells transfected with MICU1-YFP and MCU-mCherry imaged with and without the presence of histamine in 2Ca or 0Ca buffer. Images and analyses were obtained from at least 10 cells in each of 2 different experiments on 4 independent days (n = 8). Data are shown as mean +/- SEM. **P<0.05* vs. respective control conditions calculated with unpaired double sided T-Test. Source data are provided as a Source Data file.

Supplementary Figure 27:

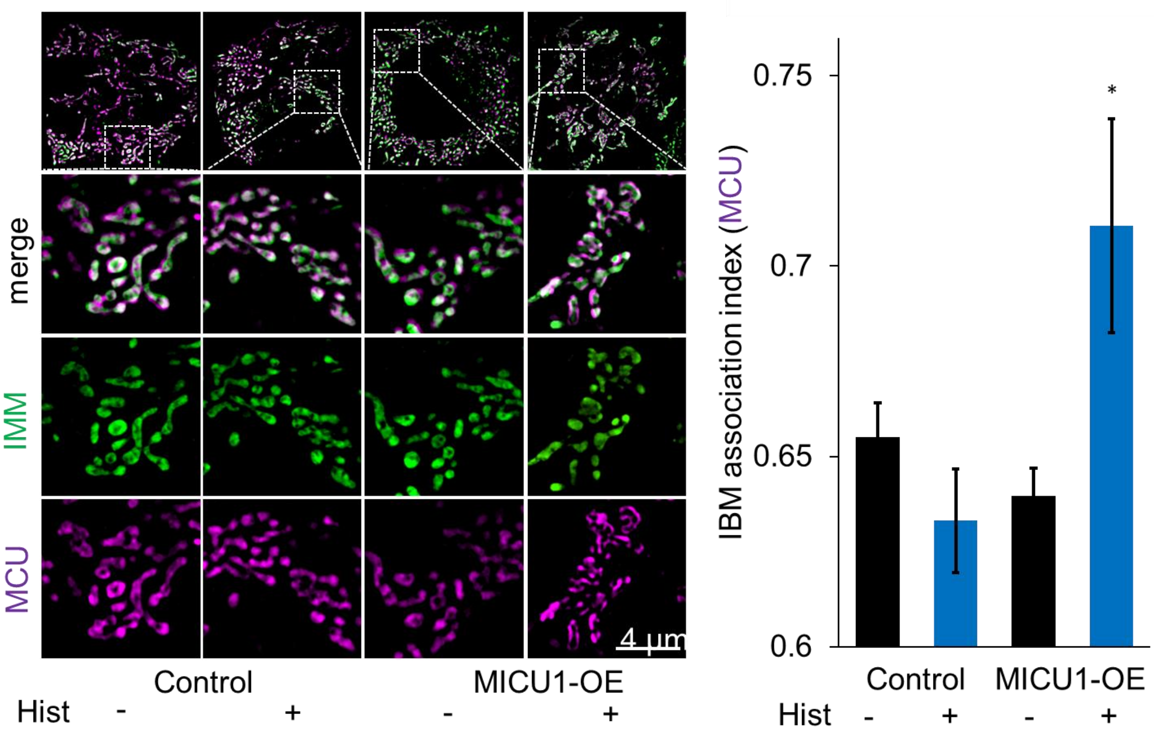

MCU IBM recruitment is dependent on MICU1. Representative Images of HeLa cells transfected with MCU-mCherry, stained with MTG and transfected with or without MICU1-OE vector imaged with and without the presence of histamine. Images and analyses were obtained from at least 10 cells in each of 2 different experiments on 4 independent days (n = 8). Data are shown as mean +/- SEM. *$P<0.05$ vs. respective control conditions calculated with unpaired double sided T-Test. Source data are provided as a Source Data file.

Supplementary Figure 28:

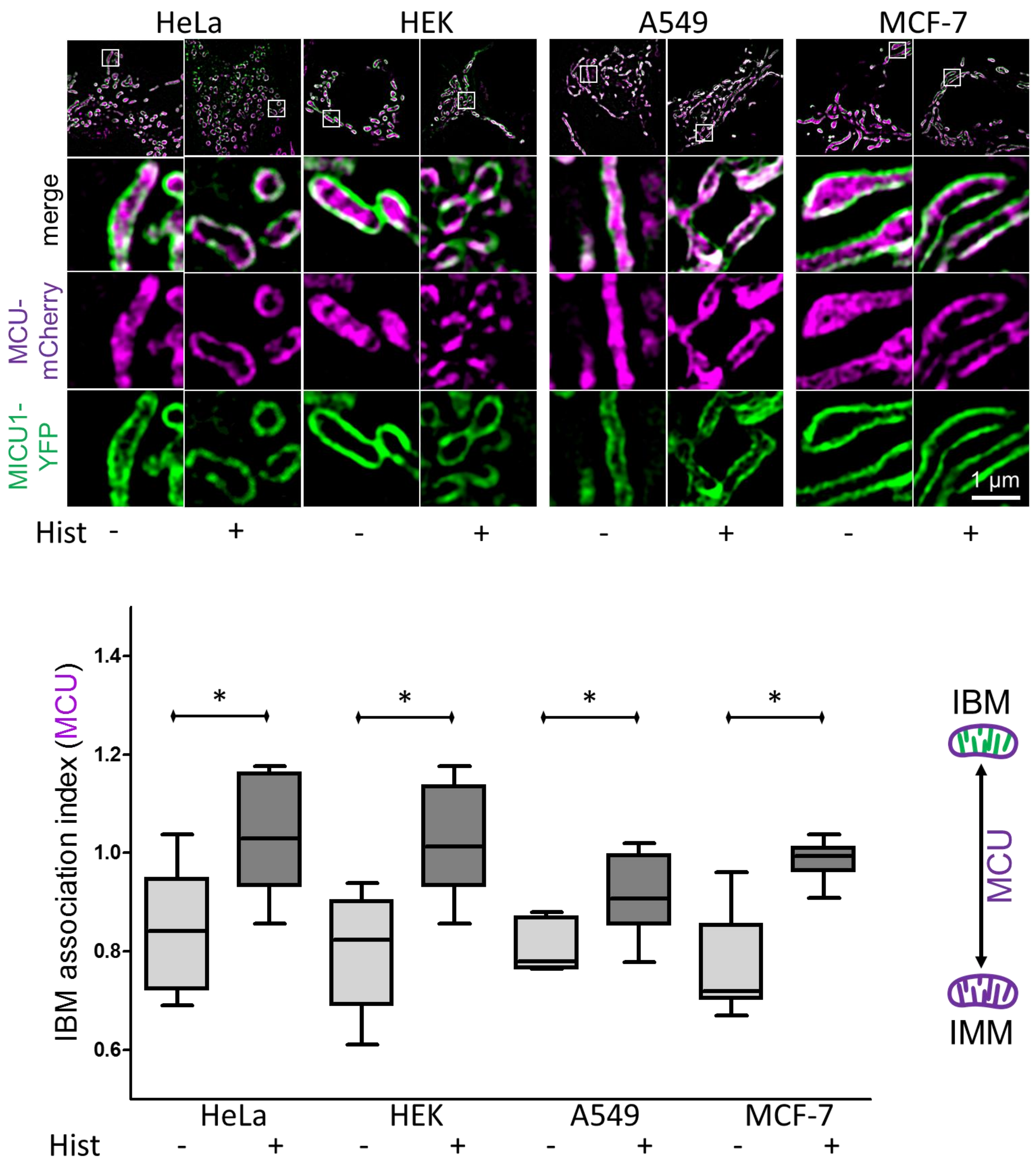

MCU-shuttling in non-excitable HeLa, HEK, A549, and MCF-7 cells. Representative images and IBM association factor of HeLa, HEK, A549, or MCF-7 cells transfected with MCU-mCherry and MICU1-YFP treated with and without histamine. Images and analyses were obtained from n = 8 independent experiments, which assessed a total of 40 cells. Horizontal lines represent the median, the lower and upper hinge show respectively first quartile and third quartile, and lower and upper whisker encompass minimal and maximal values. * = $P<0.05$ vs.

respective control conditions calculated with unpaired double sided T-Test. Source data are provided as a Source Data file.

Supplementary Figure 29:

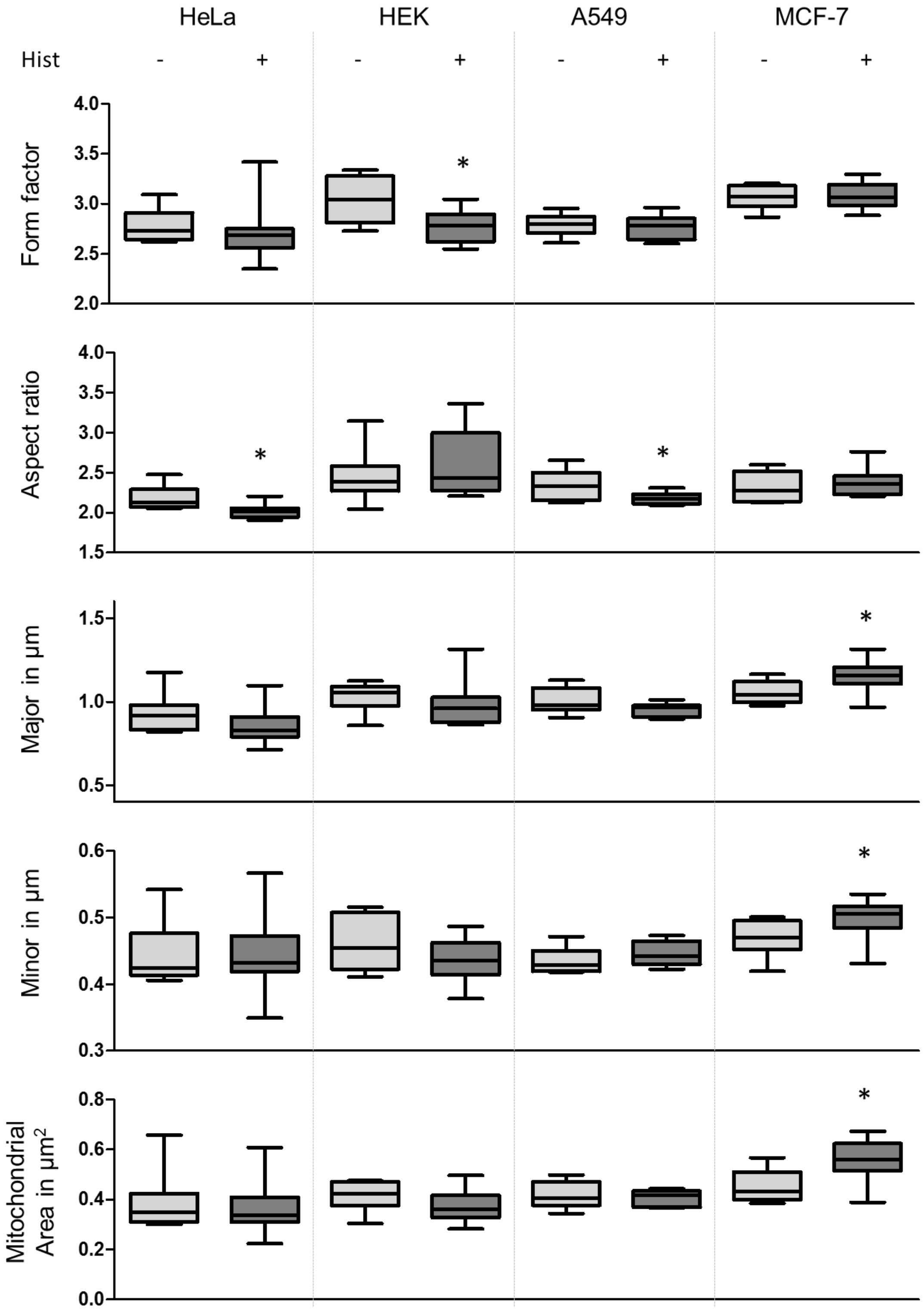

Mitochondrial morphology in non-excitable HeLa, HEK, A549, and MCF-7 cells. HeLa, HEK, A549, or MCF-7 cells were transfected with MCU-mCherry and MICU1-YFP and treated with and without histamine. The MICU1-YFP channel was used to analyze mitochondrial form factor, aspect ratio, major/minor diameters as well as the mitochondrial area. Analyses were

obtained from n = 8 independent experiments, which assessed a total of 40 cells. Horizontal lines represent the median, the lower and upper hinge show respectively first quartile and third quartile, and lower and upper whisker encompass minimal and maximal values. * = $P<0.05$ vs. respective control conditions carried out with analysis of variance (ANOVA) with Bonferroni post-hoc test. Source data are provided as a Source Data file.

Supplementary Figure 30:

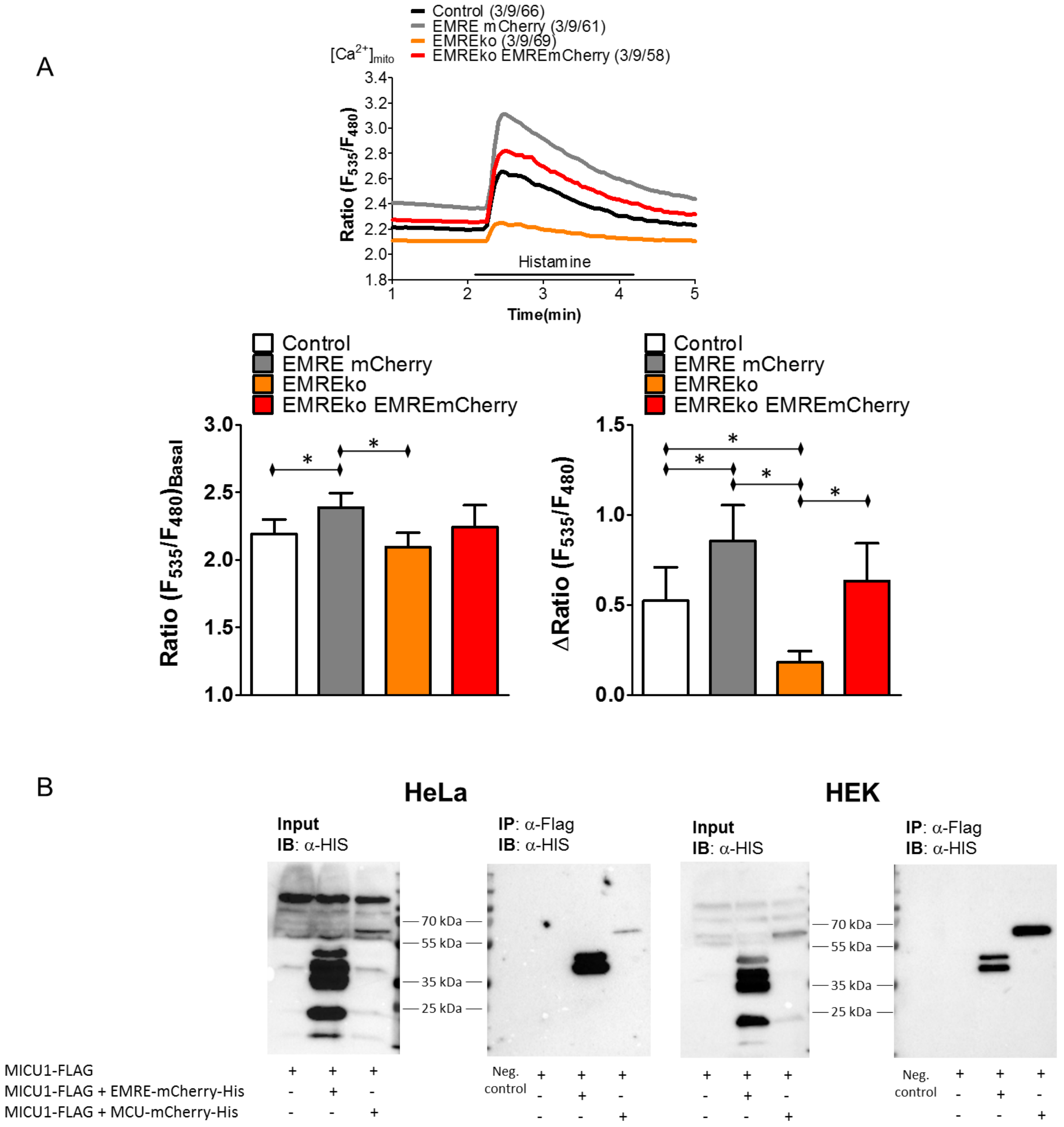

EMRE-mCherry is functional active and binds to MICU1. (A) HeLa cells and HeLa-EMRE-KO cells were transfected with the mitochondrial matrix Ca^{2+} sensor 4mt3cpv and with or without EMRE-mCherry. The basal and maximal delta Ca^{2+} signals induced by histamine were analyzed. Analyses were obtained from 3 different experiments on 3 independent days (n = 9). Data are shown as mean +/- SD. (B) HeLa or HEK cells were transfected with MICU1-FLAG alone or in combination with MCU-mCherry-His or EMRE-mCherry-His. MICU1 was immunoprecipitated using the FLAG-tag from whole cell lysate and the co-precipitated proteins were immunoblotted with a His-antibody (n = 3).*P<0.05 vs. respective control conditions

carried out with analysis of variance (ANOVA) with Bonferroni post-hoc test. Source data are provided as a Source Data file.

Supplementary Figure 31:

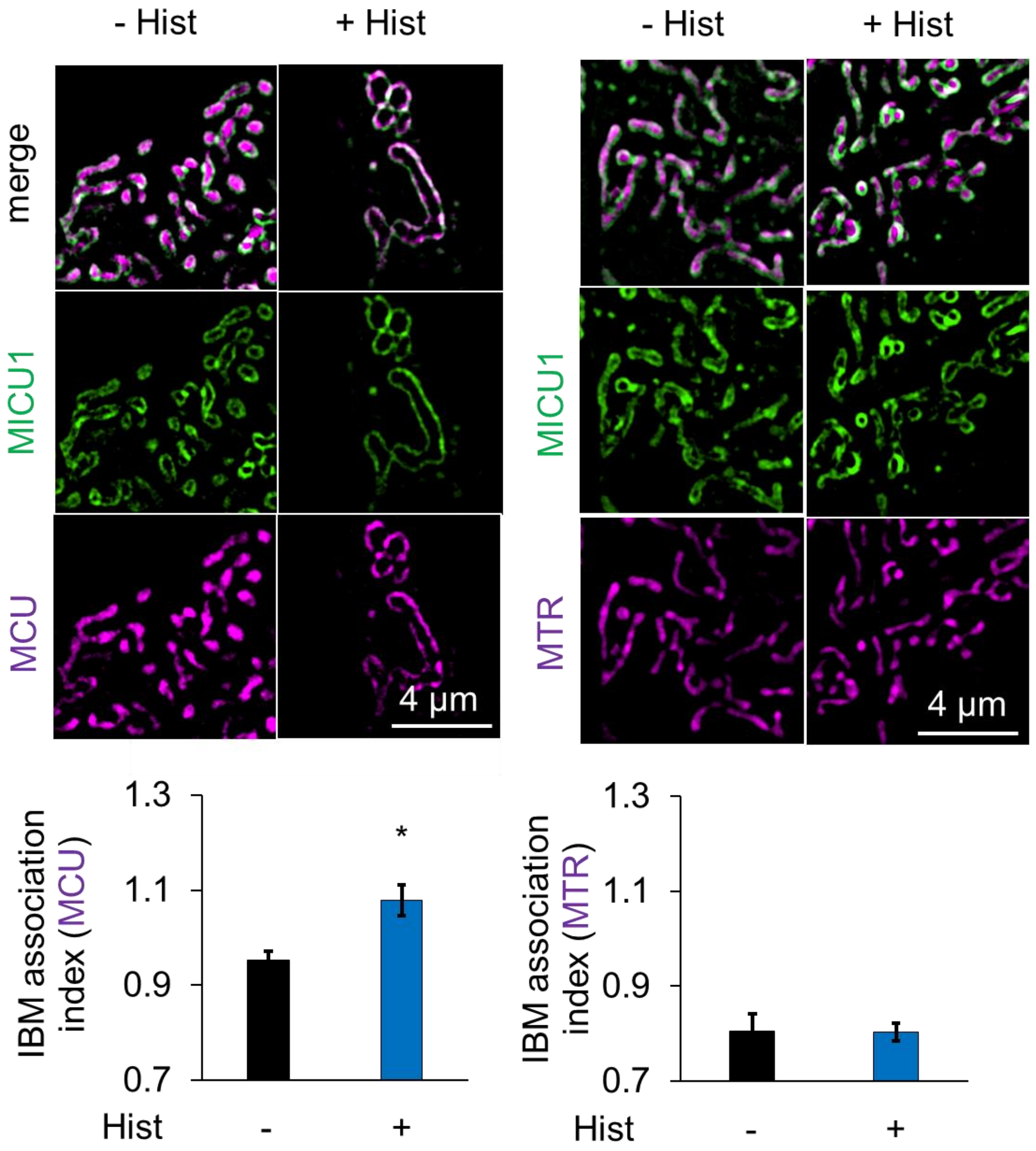

The integrity of the IMM is not disrupted by MICU1-YFP expression in either resting or histamine-stimulated cells. Representative images of HeLa cells transfected with MICU1-YFP and MCU-mCherry or stained with MTR and imaged in the absence or presence of histamine. The sub-mitochondrial localization was determined. Images and analyses were obtained from n = 8 independent experiments, which assessed a total of 40 cells. Data are

shown as mean +/- SEM. * = $P<0.05$ vs. respective control conditions calculated with unpaired double sided T-Test. Source data are provided as a Source Data file.

Supplementary Figure 32:

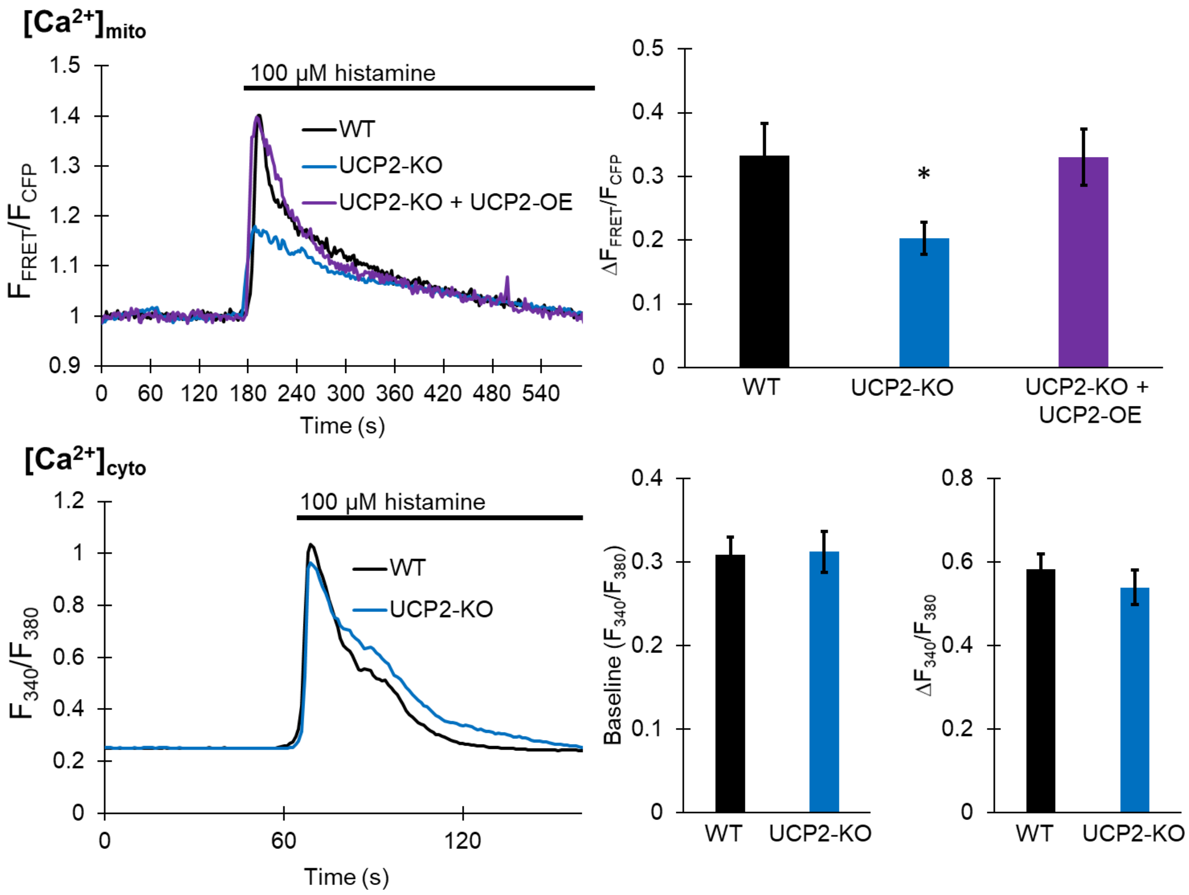

Mitochondrial Ca^{2+} concentrations are decreased while cytosolic Ca^{2+} signals upon histamine stimulation are not altered in HeLa UCP-KO cells. (upper panel) HeLa or HeLa UCP2-KO cells were transfected with 4mt3cpv while HeLa UCP2-KO cells were co transfected with or without an UCP2-expression plasmid. Cells were treated with 100 µM histamine and mitochondrial Ca^{2+} signals were recorded over time. The upper panel shows representative experiments of HeLa, HeLa UCP2-KO and the UCP2 recovery in HeLa UCP2-KO cells. Bars represent maximal Δ FRET ratio values (mean ± SEM) extracted from curves ($n_{Control}$ = 11; $n_{UCP2\text{-}KO}$ = 18; $n_{UCP2\text{-}KO/UCP2\text{-}OE}$ = 12). (lower panel) HeLa or HeLa UCP2-KO cells were incubated with Fura-2 and cytosolic Ca^{2+} signals were recorded upon treatment with 100 µM histamine. The lower panel shows representative experiments of HeLa and HeLa UCP2-KO cells. Bars represent baseline measurements F_{340}/F_{380} and maximal $\Delta F_{340}/F_{380}$ ratio values (mean ± SEM) extracted from curves ($n_{Control}$ = 9; $n_{UCP2\text{-}KO}$ = 9). Data are shown as mean +/- SEM. * = *P<0.05* vs. respective control conditions carried out with analysis of variance (ANOVA) with Bonferroni post-hoc test. Source data are provided as a Source Data file.

Supplementary Figure 33:

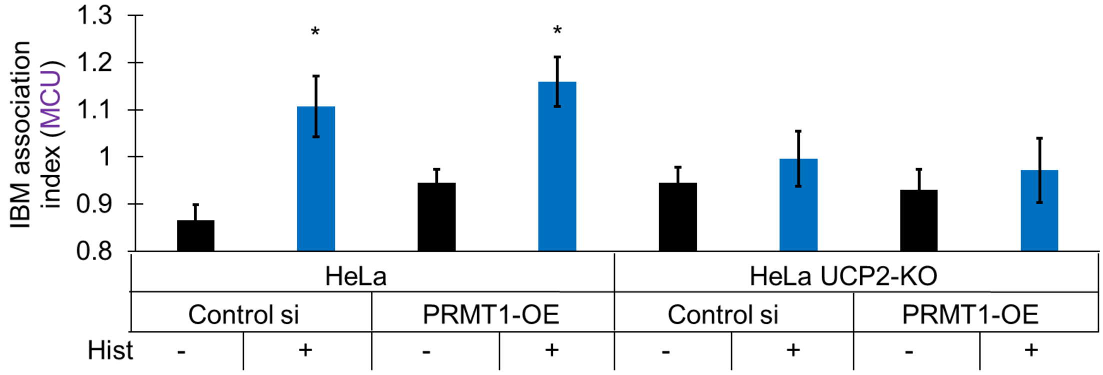

PRMT1-OE does not change the sub-mitochondrial localization of MCU in Hela cells. MICU1-YFP and MCU-mCherry were expressed in control HeLa and UCP2-KO-HeLa cells with and without PRMT1 knockdown. Sub mitochondrial localization of MCU-mCherry was analyzed with and without histamine treatment. Images and analyses were obtained from n = 8 independent experiments, which assessed a total of 40 cells. Data are shown as mean +/- SEM. * = $P<0.05$ vs. respective control conditions calculated with unpaired double sided T-Test. Source data are provided as a Source Data file.

Supplementary Figure 34:

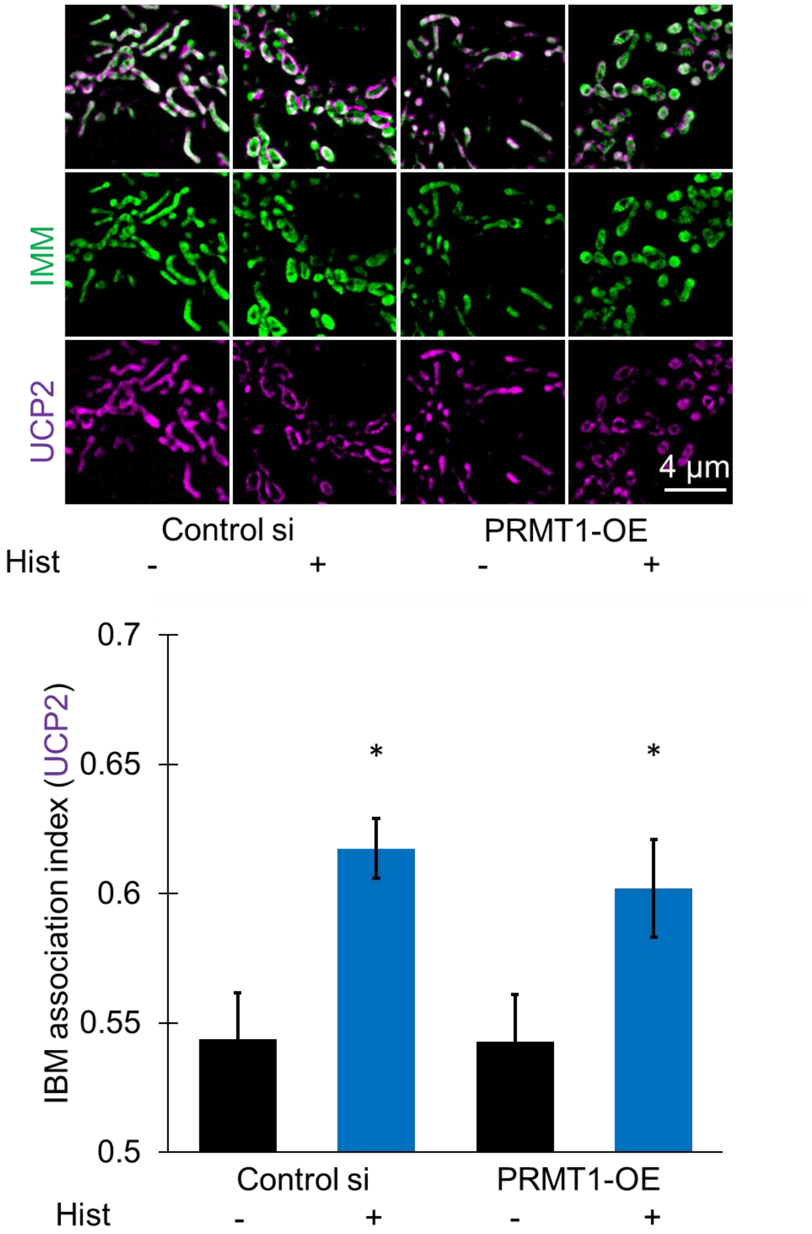

PRMT1-OE does not change the sub-mitochondrial localization of UCP2 in Hela cells. HeLa cells transfected with UCP2-mCherry with or without parallel overexpression of PRMT1 and stained with MTG. They were imaged with 3D-SIM with or without the presence of histamine. Sub mitochondrial localization of UCP2-mCherry was analyzed. Images and analyses were obtained from n = 8 independent experiments, which assessed a total of 40 cells. Data are shown as mean +/- SEM. * = *P<0.05* vs. respective control conditions calculated with unpaired double sided T-Test. Source data are provided as a Source Data file.

Supplementary Figure 35:

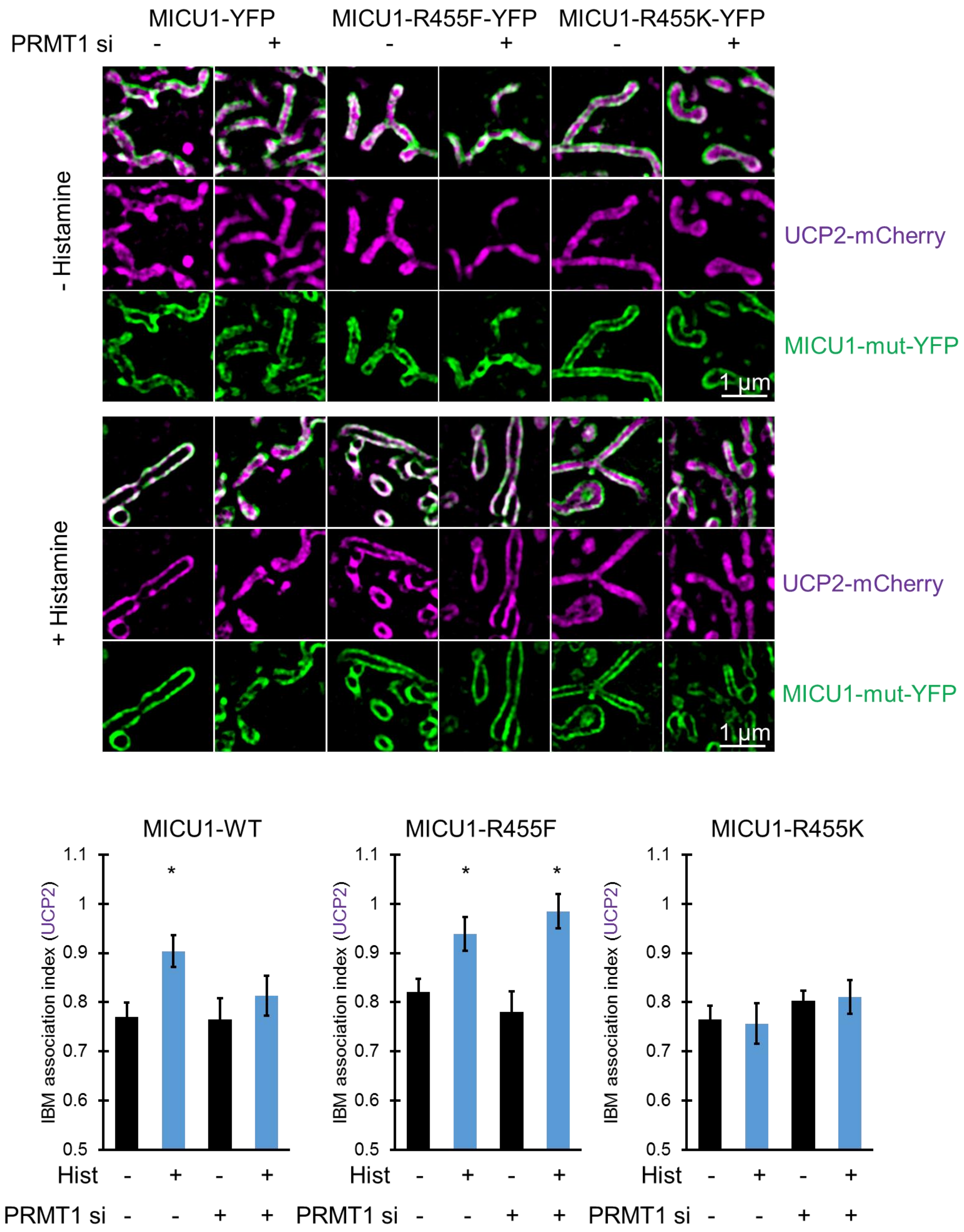

UCP2 translocation is influenced by MICU1-K and MICU1-F expression in HeLa cells. HeLa cells were transfected with UCP2-mCherry and either MICU1-YFP, or the MICU1 mutants MICU1-K-mut-YFP or MICU1-F-mut-YFP while endogenous MICU1 was knocked down with nonCDS MICU1 siRNA. Additionally cells were treated with or without PRMT1

siRNA and imaged with and without histamine treatment. The upper panel shows representative images of mitochondria under the indicated conditions. In the lower panel the quantitative analysis of UCP2-mCherry sub-mitochondrial localization in cells expressing MICU1-YFP, MICU1-K-mut-YFP or MICU1-F-mut-YFP with and without PRMT1 knockdown and with and without histamine treatment is shown. Images and analyses were obtained from at least 10 cells in each of 2 different experiments on 3 independent days (n = 6). Data are shown as mean +/- SEM. * = $P<0.05$ vs. respective control conditions calculated with unpaired double sided T-Test. Source data are provided as a Source Data file.

Supplementary Figure 36:

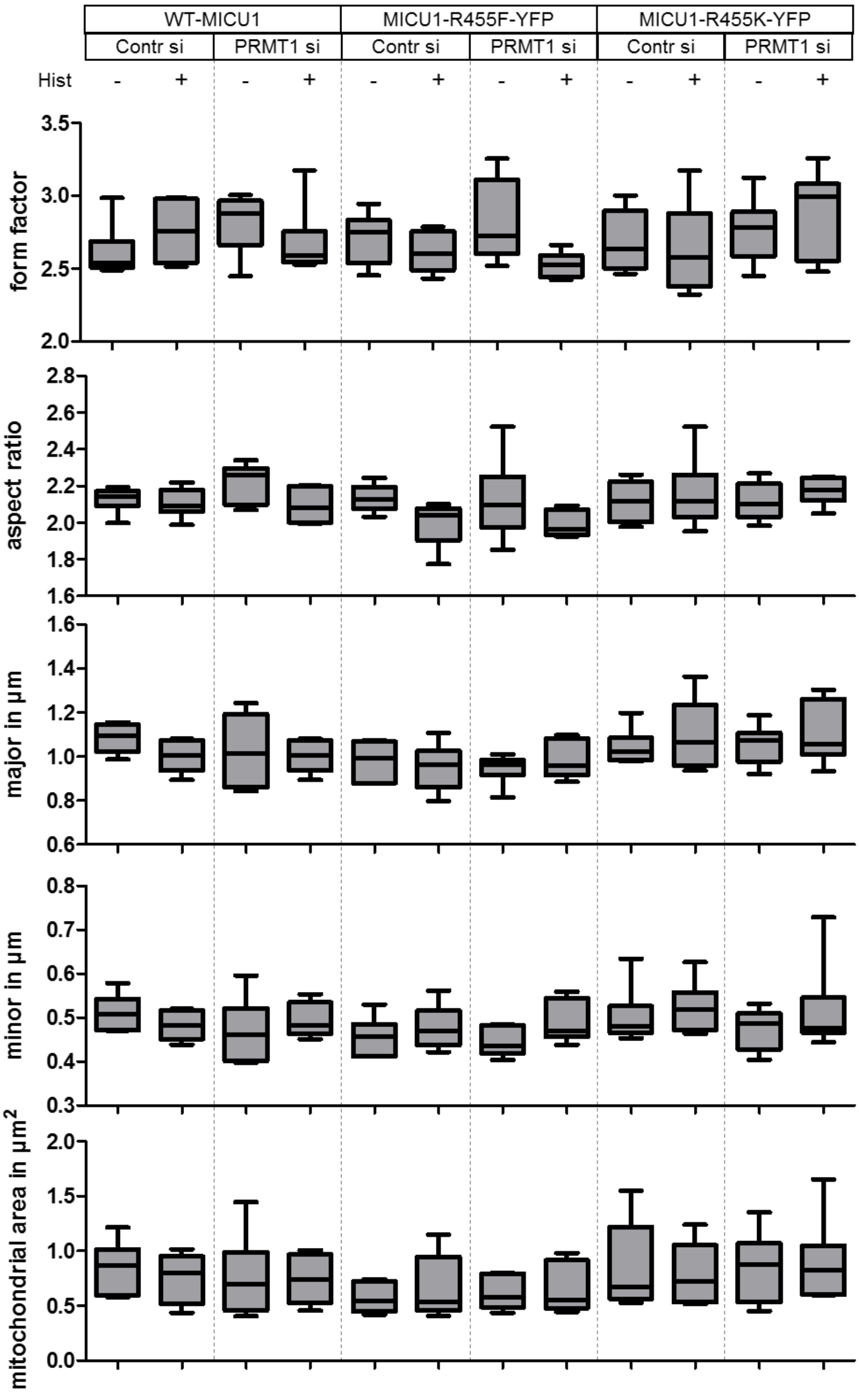

Mitochondrial morphology is not influenced by MICU1-K and MICU1-F expression in HeLa cells. HeLa cells were transfected with UCP2-mCherry and either MICU1-YFP, or the MICU1 mutants MICU1-K-mut-YFP or MICU1-F-mut-YFP while endogenous MICU1 was knocked down with nonCDS-MICU1 siRNA. Additionally, cells were treated with or without PRMT1 siRNA and imaged with and without histamine treatment. The mitochondrial form factor, aspect ratio, major/minor diameters as well as mitochondrial area were measured. Analyses were obtained from at least 10 cells in each of 2 different experiments on 3

independent days (n = 6). Horizontal lines represent the median, the lower and upper hinge show respectively first quartile and third quartile, and lower and upper whisker encompass minimal and maximal values. * = $P<0.05$ vs. respective control conditions carried out with analysis of variance (ANOVA) with Bonferroni post-hoc test. Source data are provided as a Source Data file.
