# Supplementary material for: MICU1 controls cristae junction and spatially anchors mitochondrial Ca2+ uniporter complex
Source: Nat Commun. 2019 Aug 19;10:3732. doi: 10.1038/s41467-019-11692-x (PMC6700202; doi:10.1038/s41467-019-11692-x)
Supplement: Supplementary file 3 — Reporting Summary [file 41467_2019_11692_MOESM3_ESM.pdf]

nature research

Corresponding author(s): Wolfgang Graier

Last updated by author(s): Jul 23, 2019

Reporting Summary

Nature Research wishes to improve the reproducibility of the work that we publish. This form provides structure for consistency and transparency in reporting. For further information on Nature Research policies, see Authors & Referees and the Editorial Policy Checklist.

Statistics

For all statistical analyses, confirm that the following items are present in the figure legend, table legend, main text, or Methods section.

n/a	Confirmed	
☐	☒	The exact sample size (*n*) for each experimental group/condition, given as a discrete number and unit of measurement
☐	☒	A statement on whether measurements were taken from distinct samples or whether the same sample was measured repeatedly
☐	☒	The statistical test(s) used AND whether they are one- or two-sided<br>*Only common tests should be described solely by name; describe more complex techniques in the Methods section.*
☐	☒	A description of all covariates tested
☐	☒	A description of any assumptions or corrections, such as tests of normality and adjustment for multiple comparisons
☐	☒	A full description of the statistical parameters including central tendency (e.g. means) or other basic estimates (e.g. regression coefficient) AND variation (e.g. standard deviation) or associated estimates of uncertainty (e.g. confidence intervals)
☐	☒	For null hypothesis testing, the test statistic (e.g. *F*, *t*, *r*) with confidence intervals, effect sizes, degrees of freedom and *P* value noted<br>*Give P values as exact values whenever suitable.*
☒	☐	For Bayesian analysis, information on the choice of priors and Markov chain Monte Carlo settings
☒	☐	For hierarchical and complex designs, identification of the appropriate level for tests and full reporting of outcomes
☐	☒	Estimates of effect sizes (e.g. Cohen's *d*, Pearson's *r*), indicating how they were calculated

Our web collection on statistics for biologists contains articles on many of the points above.

Software and code

Policy information about availability of computer code

Data collection	NIS-elements(4.30), Visiview(2.0.8), live acquisition software (2.0.0.12)
Data analysis	ImageJ (1.50g), Microsoft Excel Professional Plus 2013, NIS-elements(4.30), Graphpad Prism (5.01)

For manuscripts utilizing custom algorithms or software that are central to the research but not yet described in published literature, software must be made available to editors/reviewers. We strongly encourage code deposition in a community repository (e.g. GitHub). See the Nature Research guidelines for submitting code & software for further information.

Data

Policy information about availability of data

All manuscripts must include a data availability statement. This statement should provide the following information, where applicable:

- Accession codes, unique identifiers, or web links for publicly available datasets
- A list of figures that have associated raw data
- A description of any restrictions on data availability

The data that support the findings of this study are available from the authors on reasonable request, see author contributions for specific data sets.All source data for the preparation of this manuscript are available from the authors on request. The source data underlying all figures 1d, 2d, 2e, 2g, 3a, 3b, 4d, 4f, 5a, 5c, 5d, 5f, 5g, 5h, 6b, 6d, 7b, 7d, 8a and 8c and supplementary figures 1, 3, 4, 5, 7, 8, 10, 11, 12, 13, 14, 15, 16, 17, 18, 19, 20, 21, 22, 23, 24, 26, 27, 28, 29, 30, 31, 32, 33, 34, 35 and 36 are provided as a Source Data file.

Field-specific reporting

Please select the one below that is the best fit for your research. If you are not sure, read the appropriate sections before making your selection.

☒ Life sciences ☐ Behavioural & social sciences ☐ Ecological, evolutionary & environmental sciences

For a reference copy of the document with all sections, see nature.com/documents/nr-reporting-summary-flat.pdf

Life sciences study design

All studies must disclose on these points even when the disclosure is negative.

Sample size	No statistical methods was used to predetermine the sample size.
Data exclusions	No Data exclusions were done.
Replication	All attempts at replication were successful.
Randomization	Randomization was not relevant to our study.
Blinding	Due to the workflow of sample acquisition and analysis blinding was not relevant to our study.

Reporting for specific materials, systems and methods

We require information from authors about some types of materials, experimental systems and methods used in many studies. Here, indicate whether each material, system or method listed is relevant to your study. If you are not sure if a list item applies to your research, read the appropriate section before selecting a response.

Materials & experimental systems

n/a	Involved in the study
☐	☒ Antibodies
☐	☒ Eukaryotic cell lines
☒	☐ Palaeontology
☒	☐ Animals and other organisms
☒	☐ Human research participants
☒	☐ Clinical data

Methods

n/a	Involved in the study
☒	☐ ChIP-seq
☒	☐ Flow cytometry
☒	☐ MRI-based neuroimaging

Antibodies

Antibodies used

anti-Cytochrome c antibody (Cell Signaling, 12963)
Alexa488-labeled goat anti-mouse IgG (Invitrogen, A11001)
anti-Flag M2 affinity gel (Sigma-Aldrich; A2220-1ML)
monoclonal mouse anti-6x His Tag antibody (Thermo Fisher; MA1-21315)
HRP-conjugated immunoblot reagent Veriblot (Abcam; ab131366)
HRP-conjugated goat-anti-mouse secondary antibody (Thermo Fisher; 31430)
polyclonal rabbit anti-MICU1 (Cell Signaling; 12524S)
primary antibody against β-actin (Sigma, A5316)

Validation

anti-Cytochrome c antibody (Cell Signaling, 12963):
Cytochrome c (6H2.B4) Mouse mAb recognizes endogenous levels of total cytochrome c protein by immunofluorescence. This antibody is not recommended for western blot.
Species Reactivity:
Human, Mouse, Rat
Citations:
1. Metformin reveals a mitochondrial copper addiction of mesenchymal cancer cells.
In PLoS ONE on 7 November 2018 by Müller, S., Versini, A., et al..
Applications: IF (1:500)
Reactivity: None Available
2. Salinomycin kills cancer stem cells by sequestering iron in lysosomes.
In Nature Chemistry on 1 October 2017 by Mai, T. T., Hamaï, A., et al..
Applications IF (1:200)
Reactivity: Homo sapiens (Human)
3. MIR494 reduces renal cancer cell survival coinciding with increased lipid droplets and mitochondrial changes.
In BMC Cancer on 21 January 2016 by Dutta, P., Haller, E., et al..

Applications IF (1:250)
Reactivity: Homo sapiens (Human)

Alexa488-labeled goat anti-mouse IgG (Invitrogen, A11001):
Product Specific InformationTo minimize cross-reactivity, these goat anti-mouse IgG whole antibodies have been cross-adsorbed against human IgG and human serum. Cross-adsorption or pre-adsorption is a purification step to increase specificity of the antibody resulting in higher sensitivity and less background staining. The secondary antibody solution is passed through a column matrix containing immobilized serum proteins from potentially cross-reactive species. Only the nonspecific-binding secondary antibodies are captured in the column, and the highly specific secondaries flow through. The benefits of this extra step are apparent in multiplexing/multicolor-staining experiments (e.g., flow cytometry) where there is potential cross-reactivity with other primary antibodies or in tissue/cell fluorescent staining experiments where there are may be the presence of endogenous immunoglobulins.
Citations:
1. Frontiers in microbiology
HELZ2 Is an IFN Effector Mediating Suppression of Dengue Virus.
"A11001 was used in immunocytochemistry to identify and evaluate interferon antiviral effector genes"
Authors: Fusco DN,Pratt H,Kandilas S,Cheon SS,Lin W,Cronkite DA,Basavappa M,Jeffrey KL,Anselmo A,Sadreyev R,Yapp C,Shi X,O'Sullivan JF,Gerszten RE,Tomaru T,Yoshino S,Satoh T,Chung RT
2. International journal of biological sciences
Regulatory Axis of miR-195/497 and HMGA1-Id3 Governs Muscle Cell Proliferation and Differentiation.
"A11001 was used in immunocytochemistry to identify microRNAs regulating gene expression associated with muscle development"
Authors: Qiu H,Zhong J,Luo L,Tang Z,Liu N,Kang K,Li L,Gou D
3. Nature protocols
Generation of multipotent induced cardiac progenitor cells from mouse fibroblasts and potency testing in ex vivo mouse embryos.
"A11001 was used in immunocytochemistry to develop a protocol to generate expandable and multipotent induced cardiac progenitor cells from mouse adult fibroblasts"
Authors: Lalit PA,Rodriguez AM,Downs KM,Kamp TJ

anti-Flag M2 affinity gel (Sigma-Aldrich; A2220-1ML):
Anti-FLAG M2 Affinity gel is a mouse monoclonal antibody that is covalently attached to agarose. The antibody binds FLAG at the N-terminal, Met-N-terminal, C-terminal and internal locations of fusion proteins. Binding is calcium-independent.
1. Substrate specificity and effect on GLUT4 translocation of the Rab GTPase-activating protein Tbc1d1
William G. Roach, Jose A. Chavez, Cristinel P. Mîinea, Gustav E. Lienhard
Biochem J. 2007 Apr 15; 403(Pt 2): 353–358. Prepublished online 2007 Feb 2. Published online 2007 Mar 26. doi: 10.1042/BJ2006179
2. TAZ Promotes Cell Proliferation and Epithelial-Mesenchymal Transition and Is Inhibited by the Hippo Pathway
Qun-Ying Lei, Heng Zhang, Bin Zhao, Zheng-Yu Zha, Feng Bai, Xin-Hai Pei, Shimin Zhao, Yue Xiong, Kun-Liang Guan
Mol Cell Biol. 2008 Apr; 28(7): 2426–2436. Published online 2008 Jan 28. doi: 10.1128/MCB.01874-07
3. Alternative splicing regulates the expression of G9A and SUV39H2 methyltransferases, and dramatically changes SUV39H2 functions
Oriane Mauger, Roscoe Klinck, Benoit Chabot, Christian Muchardt, Eric Allemand, Eric Batsché
Nucleic Acids Res. 2015 Feb 18; 43(3): 1869–1882. Published online 2015 Jan 20. doi: 10.1093/nar/gkv013

monoclonal mouse anti-6x His Tag antibody (Thermo Fisher; MA1-21315):
1. An inhibitory mono-ubiquitylation of the Drosophila initiator caspase Dronc functions in both apoptotic and non-apoptotic pathways
Hatem Elif Kamber Kaya, Mark Ditzel, Pascal Meier, Andreas Bergmann
PLoS Genet. 2017 Feb; 13(2): e1006438. Published online 2017 Feb 16. doi: 10.1371/journal.pgen.1006438
2. Tyrosine 601 of Bacillus subtilis DnaK Undergoes Phosphorylation and Is Crucial for Chaperone Activity and Heat Shock Survival
Lei Shi, Vaishnavi Ravikumar, Abderahmane Derouiche, Boris Macek, Ivan Mijakovic
Front Microbiol. 2016; 7: 533. Published online 2016 Apr 19. doi: 10.3389/fmicb.2016.00533
3. Autophagy enhances NFκB activity in specific tissue macrophages by sequestering A20 to boost antifungal immunity
Masashi Kanayama, Makoto Inoue, Keiko Danzaki, Gianna Hammer, You-Wen He, Mari L. Shinohara
Nat Commun. 2015; 6: 5779. Published online 2015 Jan 22. doi: 10.1038/ncomms6779

HRP-conjugated immunoblot reagent Veriblot (Abcam; ab131366):
1. Anti-tumor effects of mAb against l-type amino acid transporter 1 (LAT1) bound to human and monkey LAT1 with dual avidity modes
Shiho Ueda, Hidemi Hayashi, Takako Miyamoto, Shinya Abe, Kana Hirai, Kanji Matsukura, Hideki Yagi, Yuta Hara, Kinji Yoshida, Shogo Okazaki, Masakazu Tamura, Yuki Abe, Toshinori Agatsuma, Shin-ichiro Niwa, Kazue Masuko, Takashi Masuko
Cancer Sci. 2019 Feb; 110(2): 674–685. Published online 2019 Jan 4. doi: 10.1111/cas.13908
2. Targeted Delivery of Stk25 Antisense Oligonucleotides to Hepatocytes Protects Mice Against Nonalcoholic Fatty Liver Disease
Emmelie Cansby, Esther Nuñez-Durán, Elin Magnusson, Manoj Amrutkar, Sheri L. Booten, Nagaraj M. Kulkarni, L. Thomas Svensson, Jan Borén, Hanns-Ulrich Marschall, Mariam Aghajan, Margit Mahlapuu
Cell Mol Gastroenterol Hepatol. 2019; 7(3): 597–618. Published online 2018 Dec 19. doi: 10.1016/j.jcmgh.2018.12.004
3. ZNF506-dependent positive feedback loop regulates H2AX signaling after DNA damage
Somaira Nowsheen, Khaled Aziz, Kuntian Luo, Min Deng, Bo Qin, Jian Yuan, Karthik B. Jeganathan, Jia Yu, Henan Zhang, Wei Ding, Jan M. van Deursen, Zhenkun Lou
Nat Commun. 2018; 9: 2736. Published online 2018 Jul 16. doi: 10.1038/s41467-018-05161-0

HRP-conjugated goat-anti-mouse secondary antibody (Thermo Fisher; 31430):

Product # 31430 has been successfully used in Western blot, IHC and IP applications.
Product # 31430 reacts with the heavy chains of mouse igg and with the light chains common to most mouse immunoglobulins, but does not react against non-immunoglobulin serum proteins. However, this antibody may cross-react with immunoglobulins from other species.
1. Acute death of astrocytes in blast-exposed rat organotypic hippocampal slice cultures
Anna P. Miller, Alok S. Shah, Brandy V. Aperi, Shekar N. Kurpad, Brian D. Stemper, Aleksandra Glavaski-Joksimovic
PLoS One. 2017; 12(3): e0173167. Published online 2017 Mar 6. doi: 10.1371/journal.pone.0173167
2. The Alzheimer's disease risk factors apolipoprotein E and TREM2 are linked in a receptor signaling pathway
Charlotte Jendresen, Vibeke Årskog, Michael R. Daws, Lars N. G. Nilsson
J Neuroinflammation. 2017; 14: 59. Published online 2017 Mar 21. doi: 10.1186/s12974-017-0835-4
3. A biomimetic gelatin-based platform elicits a pro-differentiation effect on podocytes through mechanotransduction
Mufeng Hu, Evren U. Azeloglu, Amit Ron, Khanh-Hoa Tran-Ba, Rhodora C. Calizo, Iman Tavassoly, Smiti Bhattacharya, Gomathi Jayaraman, Yibang Chen, Vera Rabinovich, Ravi Iyengar, James C. Hone, John C. He, Laura J. Kaufman
Sci Rep. 2017; 7: 43934. Published online 2017 Mar 6. doi: 10.1038/srep43934

polyclonal rabbit anti-MICU1 (Cell Signaling; 12524S):
CBARA1/MICU1 (D4P8Q) Rabbit mAb recognizes endogenous levels of total CBARA1/MICU1 protein.
Species Reactivity: Human, Mouse, Rat, Monkey
1. Exploring the In Vivo Role of the Mitochondrial Calcium Uniporter in Brown Fat Bioenergetics
Daniel Flicker, Yasemin Sancak, Eran Mick, Olga Goldberger , Vamsi K. Mootha
Cell Reports: DOI:https://doi.org/10.1016/j.celrep.2019.04.013
2. PRMT1-mediated methylation of MICU1 determines the UCP2/3 dependency of mitochondrial Ca2+ uptake in immortalized cells
Corina T. Madreiter-Sokolowski, Christiane Klec, Warisara Parichatikanond, Sarah Stryeck, Benjamin Gottschalk, Sergio Pulido, Rene Rost, Emrah Eroglu, Nicole A. Hofmann, Alexander I. Bondarenko, Tobias Madl, Markus Waldeck-Weiermair, Roland Malli, Wolfgang F. Graier
Nat Commun. 2016; 7: 12897. Published online 2016 Sep 19. doi: 10.1038/ncomms12897

primary antibody against β-actin (Sigma, A5316):
Monoclonal mouse anti-actin antibody was used as a loading control for western blot analysis of immunoprecipitated proteins from rat dorsal root ganglion cocultures.
Mouse Monoclonal Anti-β-Actin antibody has been used for western blot analyses. The antibody can also be used for immunohistochemistry, indirect immunofluorescence (1:1,000) using cultured human or chicken fibroblasts, and indirect ELISA.
Western blot analysis of MDCK cell lysates were performed using monoclonal anti-actin antibody as a primary antibody.
Monoclonal Anti-β-Actin antibody has been used in western blotting.
1. Immortalization of Primary Human Prostate Epithelial Cells by c-Myc
Jesús Gil, Preeti Kerai, Matilde Lleonart, David Bernard, Juan Cruz Cigudosa, Gordon Peters, Amancio Carnero and David Beach
Cancer Res March 15 2005 (65) (6) 2179-2185; DOI: 10.1158/0008-5472.CAN-03-403
2. Myc confers androgen-independent prostate cancer cell growth
David Bernard, Albin Pourtier-Manzanedo, Jesús Gil, David H. Beach
J Clin Invest. 2003 Dec 1; 112(11): 1724–1731. doi: 10.1172/JCI200319035
3. β-Actin specifically controls cell growth, migration, and the G-actin pool
Tina M. Bunnell, Brandon J. Burbach, Yoji Shimizu, James M. Ervasti
Mol Biol Cell. 2011 Nov 1; 22(21): 4047–4058. doi: 10.1091/mbc.E11-06-0582

Eukaryotic cell lines

Policy information about cell lines

Cell line source(s)	A-549 (CLS-300114)<br>MCF-7 (CLS-300273)<br>HeLa (ATCC-CCL-2.2TM)<br>HEK-293 (ATCC-CRL-1573TM)
Authentication	Origin of cells was confirmed by STR-profiling by the cell culture facility of ZMF (Graz).
Mycoplasma contamination	Cell lines were regularly tested for mycoplasma contamination (negative).
Commonly misidentified lines<br>(See ICLAC register)	MCF-7 and HEK-293 (both ICLAC register) cells were used to prove MCU-rearrangement upon histamine in non-excitable cells that are frequently used in the respective research area.
